# A Network Integration Approach to Predict Conserved Regulators Related to Pathogenicity of Influenza and SARS-CoV Respiratory Viruses

**DOI:** 10.1371/journal.pone.0069374

**Published:** 2013-07-25

**Authors:** Hugh D. Mitchell, Amie J. Eisfeld, Amy C. Sims, Jason E. McDermott, Melissa M. Matzke, Bobbi-Jo M. Webb-Robertson, Susan C. Tilton, Nicolas Tchitchek, Laurence Josset, Chengjun Li, Amy L. Ellis, Jean H. Chang, Robert A. Heegel, Maria L. Luna, Athena A. Schepmoes, Anil K. Shukla, Thomas O. Metz, Gabriele Neumann, Arndt G. Benecke, Richard D. Smith, Ralph S. Baric, Yoshihiro Kawaoka, Michael G. Katze, Katrina M. Waters

**Affiliations:** 1 Computational Sciences and Mathematics Division, Pacific Northwest National Laboratory, Richland, Washington, United States of America; 2 Department of Pathobiological Sciences, Influenza Research Institute, University of Wisconsin-Madison, Madison, Wisconsin, United States of America; 3 Department of Epidemiology, University of North Carolina at Chapel Hill, Chapel Hill, North Carolina, United States of America; 4 Department of Microbiology, University of Washington, Seattle, Washington, United States of America; 5 Université Pierre et Marie Curie, Centre National de la Recherche Scientifique UMR7224, Paris, France; 6 Biological Sciences Division, Pacific Northwest National Laboratory, Richland, Washington, United States of America; 7 Division of Virology, Department of Microbiology and Immunology, Institute of Medical Science, University of Tokyo, Tokyo, Japan; 8 Department of Special Pathogens, International Research Center for Infectious Diseases, Institute of Medical Science, University of Tokyo, Tokyo, Japan; 9 ERATO Infection-Induced Host Responses Project, Saitama, Japan; 10 Washington National Primate Research Center, University of Washington, Seattle, Washington, United States of America; Johns Hopkins University - Bloomberg School of Public Health, United States of America

## Abstract

Respiratory infections stemming from influenza viruses and the Severe Acute Respiratory Syndrome corona virus (SARS-CoV) represent a serious public health threat as emerging pandemics. Despite efforts to identify the critical interactions of these viruses with host machinery, the key regulatory events that lead to disease pathology remain poorly targeted with therapeutics. Here we implement an integrated network interrogation approach, in which proteome and transcriptome datasets from infection of both viruses in human lung epithelial cells are utilized to predict regulatory genes involved in the host response. We take advantage of a novel “crowd-based” approach to identify and combine ranking metrics that isolate genes/proteins likely related to the pathogenicity of SARS-CoV and influenza virus. Subsequently, a multivariate regression model is used to compare predicted lung epithelial regulatory influences with data derived from other respiratory virus infection models. We predicted a small set of regulatory factors with conserved behavior for consideration as important components of viral pathogenesis that might also serve as therapeutic targets for intervention. Our results demonstrate the utility of integrating diverse ‘omic datasets to predict and prioritize regulatory features conserved across multiple pathogen infection models.

## Introduction

**Figure 1:**
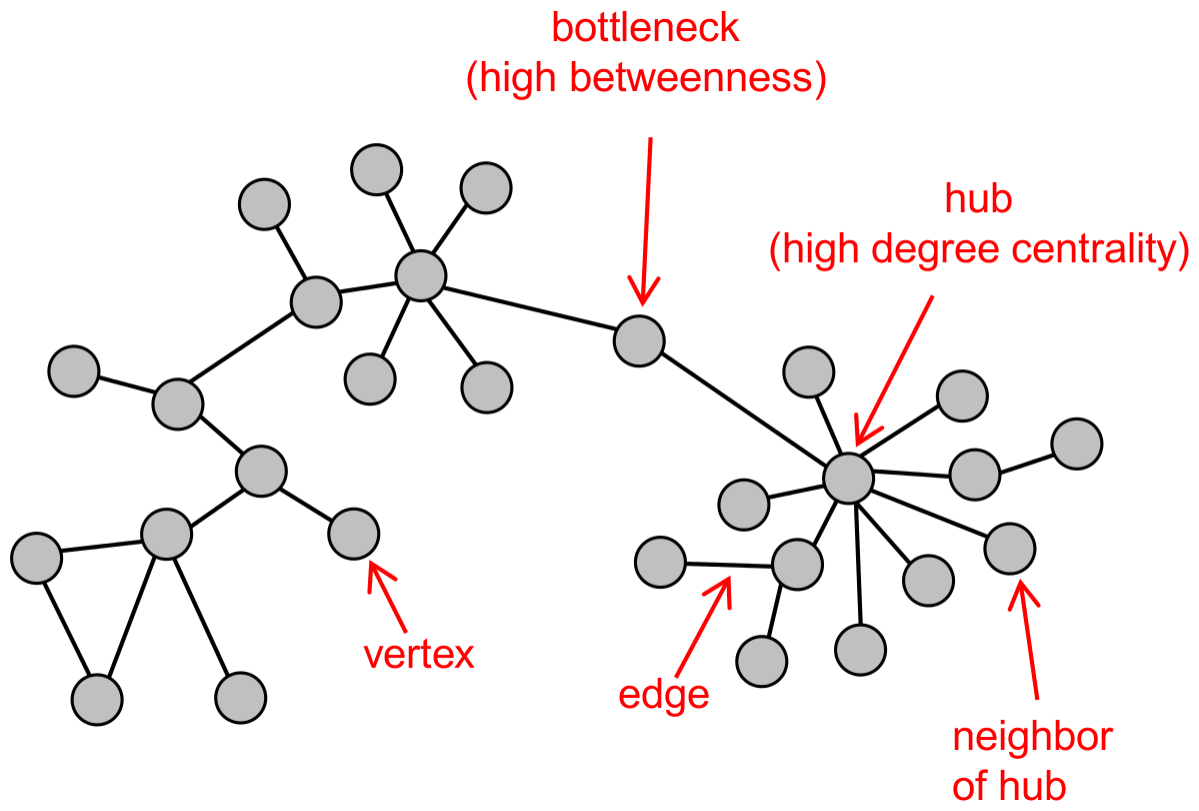
Network Terminology. Association networks capture both physical and regulatory interactions between gene pairs. Network hubs are identified by the degree centrality metric, which is the number of edges (i.e. relationships, represented by connecting lines) associated with any given vertex (elements being connected, e.g. genes, identified as circles). Network bottlenecks have high values for the betweenness centrality metric, which is the number of shortest paths between all pairs of vertices that pass through a given vertex. Network neighbors are vertices connected by a single edge.

Systems biology brings advanced bioinformatics and computational tools to bear on important health problems to identify key elements of biological processes that may function as critical signaling mediators. These predictive tools are important because global profiling methods (e.g. transcriptomics) are becoming routine approaches for examining entire systems and their response to perturbation. Data sets generated by these platforms are complex and require bioinformatics/computational tools for network reconstruction and more complex predictions of network interactions. For biological systems, network analysis has proven useful for analyzing protein-protein, protein-DNA, and kinase-substrate interactions, as well as for genetic interactions among genes, in which relationships between two genes that both contribute to a given phenotype can be seen [1]. These are fundamentally important interactions by which cells translate cellular signaling information into an appropriate biological response. More recently, advanced attempts at network reconstruction have focused on capturing regulatory associations between genes and proteins by comparing expression patterns across multiple conditions [2]–[4]. Networks of this kind are called association networks and can capture both physical interactions as well as more subtle, but equally important relationships between gene pairs or within gene clusters. Prioritization of key regulators based on network topology, which is a structural representation of the system components that includes how information flows between the parts (Figure 1), has been shown to be superior to simple ranking of differentially expressed (DE) genes [5]. Our group and others have shown that genes occupying certain topological positions within the networks frequently play important regulatory roles in biological processes [4], [6]. Two of the most studied topological features include network hubs and network bottlenecks (Figure 1). From a practical perspective, these features are often found to be important control points within a network that regulate and are connected to many important molecular processes. From a technical perspective, network hubs are identified by the degree centrality metric, which is simply the number of edges (i.e. relationships, represented by the connecting lines in Figure 1) associated with any given vertex (an element in the network, e.g. a gene, identified as circles in Figure 1). Network bottlenecks have high values for the betweenness centrality metric, which is the number of shortest paths between all pairs of vertices that pass through a given vertex. Bottlenecks appear to bridge distinct regions of a network. As yet, it remains unclear which of these topological features is the most effective predictor of regulatory function for any given network construction approach or biological context [2].

Bioinformatics is also faced with the challenge of how to best integrate multiple data types. Transcriptome data provides a read-out for gene regulation at the mRNA level, however, correlation of mRNA with its associated protein expression can be relatively low [7], [8]. Proteome data provides a complementary picture of protein expression levels; but current proteomics technologies provide only limited coverage of the proteome. Despite these limitations, integration of these discrete data types has merit and can provide significantly improved coverage of signaling networks [7]. Our group and others have developed advanced bioinformatics capabilities to facilitate the integration of diverse data types [7], [9]–[11].

**Figure 2:**
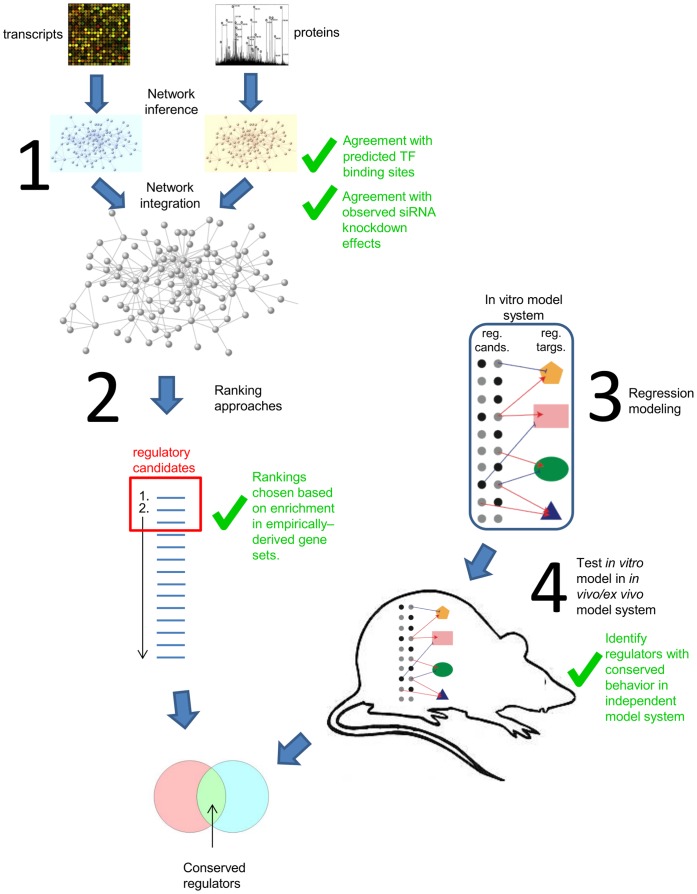
Workflow for prediction of conserved regulators. Step 1: Network inference. Network relationships are reconstructed from transcript and protein quantification data by finding similar expression patterns across multiple conditions. Protein and transcript networks are integrated to form a unified network (in the case of the SARS-CoV data; see text). Step 2: Ranking approaches. Network genes were ranked using three distinct measures: network betweenness, degree centrality, and differential expression between pathogenicity levels. Gene set enrichment analysis (GSEA) was used to test each individual ranking, and each combination of rankings, for how effectively they prioritize genes known to be relevant to viral infection. Step 3: Model construction. Multivariate regression was used to build regulatory models using the union of known transcription factors and top prioritized genes from step 2 as candidate regulators. The modeling process predicts a small set of regulatory genes that are likely to regulate each target (cluster of genes). Step 4: Cross-system comparison. Performance of the resulting models was tested in either an *in vivo* mouse model (influenza virus) or an *ex vivo* human primary lung epithelial model (SARS-CoV). *In vivo* and *ex vivo* models are both represented by the outlined mouse shape in the figure. Genes with conserved regulation in the new system were prioritized as conserved regulators for the respective virus infection (Tables 4 and 5). Green check marks indicate steps validated through comparison to/integration with outside experimental data.

In this study, we use a network approach to predict critical signaling regulators to influenza virus and severe acute respiratory syndrome coronavirus (SARS-CoV) infection. These respiratory viruses are crucial public health concerns, and the essential mechanisms behind pathogenesis are not well understood. We have generated large-scale time courses of transcriptome and proteome data derived from influenza virus and SARS-CoV infected human bronchial epithelial cells (Calu-3 cells). Our datasets include high and low pathogenicity (HP and LP) strains of both influenza and SARS-CoV viruses, based on published studies in mouse models [12], [13]. A recent study showed that building a consensus network from multiple inference algorithms yielded a better performing network than any network arising from individual algorithms [14]. Here we apply a related concept and use an analysis workflow consisting of 1) network inference, 2) a combination of network topology measures and differential expression ranking metrics to predict the best-performing ranking of key regulators, 3) regression analysis to identify regulatory relationships, and 4) comparison of model predictions from the in vitro model with other systems to identify conserved behavior (Figure 2). This procedure produced a highly prioritized list of regulators with conserved behavior for each virus. We anticipate that the genes resulting from this combined analysis will provide a valuable resource for future experimental validation studies leading to potential therapeutic intervention.

## Results

### Creation of Data Compendium

For experiments with SARS-CoV, Calu3 cells were infected with either wild type (WT) virus, a mutant strain that does not express the accessory protein open reading frame 6 (delta ORF6) [15], [16], or a SARS-like bat coronavirus containing the human SARS-CoV spike receptor binding domain (Bat-SRBD) [12], over a 72 hour time course. For the purposes of our study, we considered the wild type as HP and the two mutants as LP based on previously published studies in mouse models [12], [13]. For influenza virus, we infected cells with A/Vietnam/1203/2004 (H5N1; referred to as VN1203) for a time course of 3 to 24 hours; or A/California/04/2009 (H1N1; referred to as CA04) or A/Netherlands/602/2009 (H1N1, referred to as NL602) for a time course from 3 to 48 hours. VN1203 is a HP H5N1 avian influenza virus, while the other two strains are low pathogenic 2009 pandemic influenza viruses based on both *in vitro* cell viability and *in vivo* survival data. Microarray and proteomic data were collected and processed for all viral strains and conditions as detailed in Materials and Methods. Compendia of all data for each virus type were formed and used for all subsequent analysis. After applying fold change and significance filters (see Methods), SARS-CoV virus transcriptomics were reduced to 8695 differentially expressed (DE) genes (relative to time-matched mocks). For influenza data, we applied the fold change filter to time points at or before 12 hours, since genes affected at later time points were previously shown to be primarily involved in cell death and not regulatory events [17], [18]. This filtering scheme resulted in 13789 DE genes in influenza virus-infected cells. Proteomic analysis (see Methods) resulted in 859 and 1529 DE proteins for SARS-CoV and influenza viruses, respectively.

### Network Inference

**Figure 3:**
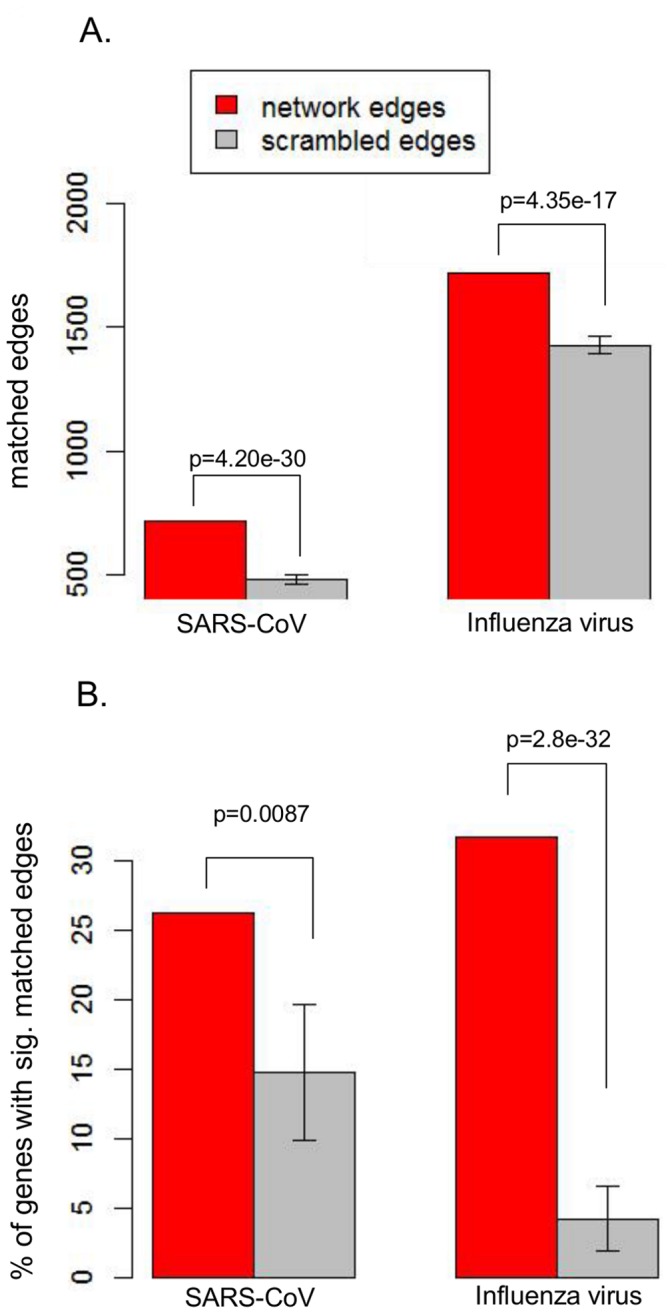
Inferred network edge validation. A) Network edges were compared to a predicted transcription factor – target database. The number of transcriptome network edges for each virus that was also present in the database (red) was compared with the number of matching edges in 1000 random networks (gray) to estimate the number of matching edges expected from chance. B) Relationships between genes targeted in a large siRNA-targeting study [19] and the downstream affected genes were compared to relationships predicted from our network inference approach. Results show the number of genes that exhibited statistically significant overlap between their network neighbors and perturbed genes from the siRNA targeting study. Red designates the overlap with neighbors from the actual network; grey designates overlap with neighbors from 500 random networks (see Materials and Methods). Error bars represent standard deviation of the distribution of gene percentages with significant overlaps.

We used a network approach to predict genes that regulate the host response to viral infection based on their topological position. First, the Context Likelihood of Relatedness (CLR) algorithm [3] was used to infer individual transcriptome and proteome networks independently for both the SARS-CoV and influenza compendia (first stage of step #1 in Figure 2). As an initial assessment of our transcriptomic networks, we determined whether the networks contained known edges based on transcription factor (TF)-target interactions in a significantly greater proportion than would be expected to arise by chance. To determine the expected number of randomly occurring edges, we performed 1000 iterations of scrambling the parent vertices for all edges in the networks (see Methods). The resulting vertices were matched to computed TF-target pairs from the TRANSFAC dataset available through the molecular signatures database (msigdb, found at http://www.broadinstitute.org/gsea/msigdb/collections.jsp). The inferred transcriptome networks contained significantly more known regulatory edges for SARS-CoV and influenza viruses than seen from randomly scrambled edges (as calculated by z-test, Figure 3a).

As an additional validation of the network inference approach, we utilized an available dataset derived from 400 siRNA knockdown experiments in human umbilical vein endothelial cells (HUVEC) [19]. We reasoned that network neighbors (or genes with a predicted relationship) should have regulatory influence on one another, such that the set of genes affected by siRNA targeting of a particular gene should be a similar set of genes to those neighboring the target in an association network. The results show that for a significant number of genes there is significant overlap between the set of differentially expressed genes when the gene is knocked down and the set of network neighbors of that same gene (Figure 3B). The percentage of these genes with significant overlap was compared to results found by comparison to random networks. Thus the networks inferred from the transcriptome compendia clearly provide predictive power.

### Network Integration

Since the proteome data covers only a fraction of the gene products of the transcriptome data platform, the resulting proteome network was much smaller than the transcriptome network, as would be expected. We therefore integrated the two data types into a single network, as demonstrated previously [7], [11]. We initially merged all of the vertices and edges for the networks, yielding two integrated networks, one for each virus. However, when we ordered all vertices according to their betweenness scores, we identified artificially high betweenness scores for vertices that happen to be common in both datasets, which is unrelated to their true betweenness in the regulatory network (Figure S1-top). To alleviate this potential artifact in the integrated networks, we selected proteome edges for integration in which both parent vertices were already present in the transcriptome network (i.e., “conservative integration”; Figure S2). In this way, only new edges (566 for influenza virus, 285 for SARS-CoV), not new vertices, were introduced into the network so that no artificial bottlenecks were introduced (Figure S1, bottom). No major changes in degree centrality (hubs) were observed as a result of integration (data not shown).

We determined the effect of proteome data integration on network bottlenecks by comparing the difference in betweenness for all vertices before and after integration. We found that proteome data integration had minimal effect on the influenza network, since the top 200 vertices in the integrated network showed only minor shifts in betweenness ranking (less than 30 positions, with 98% less than 5 positions). Further influenza virus network analysis therefore included only transcriptome data. However, there were vertices in the top 200 genes of the SARS-CoV betweenness ranking that showed large jumps in ranking positions. Table 1 shows the 8 genes with a greater than 1000 position shift into the top 200 ranked betweenness genes. Six of these have been previously shown to be associated with SARS/virus infection (Table 1), thus demonstrating the advantage of proteomics integration. Thus for SARS-CoV, an integrated transcriptome/proteome network was used for further studies.

**Table 1:** SARS genes whose topology is significantly altered by proteome data integration.

Symbol	Uniprot ID	Entrez ID	Reference to involvement in SARS/virus infection
STAT1	STAT1_HUMAN	6772	[64]
B2M	B2MG_HUMAN	567	[65]
HSPA1A	HSP71_HUMAN	3303	[66]
MIF	MIF_HUMAN	4282	[67]
WARS	SYWC_HUMAN	7453	
Annexin A4	ANXA4_HUMAN	307	[68]
HIST1H1E	H14_HUMAN	3008	
DNAJB1	DNAJB1_HUMAN	3337	[69]
KRT23	K1C23_HUMAN	25984	

### Regulatory Candidate Ranking

**Figure 4:**
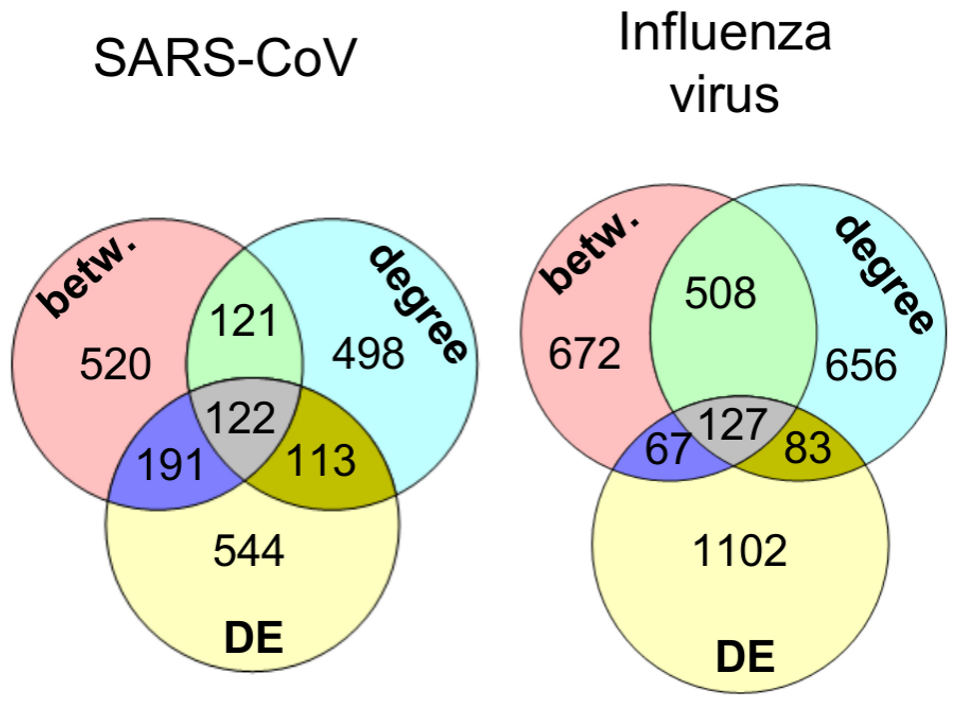
Overlap between rankings. Genes were ranked according to betweenness, degree and differential expression (DE) as described in Materials and Methods. Venn diagrams indicate the overlap in the top 10% of each of these rankings for both viruses as indicated.

We next desired to use network topology and DE to predict regulators of the infection process (step #2 in Figure 2). While both hubs and bottlenecks have been shown to represent important network vertices in various contexts [4]–[6], [20]–[22], it was unclear whether both represented important regulatory elements in our system. This question would be largely irrelevant if hubs and bottlenecks from the networks were found to represent the same group of genes. Additionally, while network topology allows prediction of regulatory elements in a biological system, they do not necessarily address pathogenicity per se. For this reason, genes were also ranked according to their overall differential expression between HP and LP strains in SARS-CoV and influenza virus infections. By comparing the top 10% within each ranking metric, we confirmed that hubs, bottlenecks and DE genes from our networks represent distinct, but not wholly independent groups (Figure 4).

**Figure 5:**
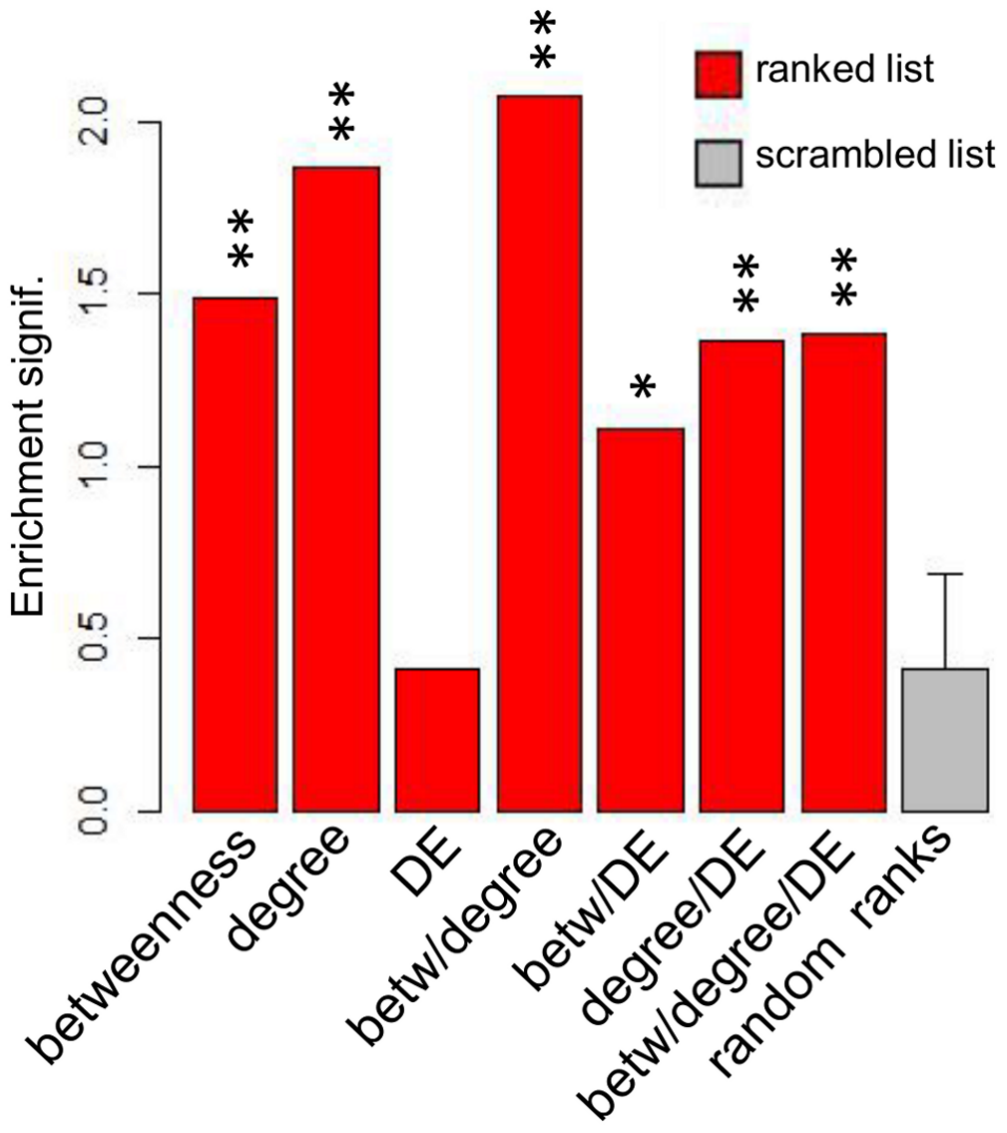
GSEA-based enrichment analysis of influenza rankings. Seven distinct rankings of genes from the influenza network were evaluated for their enrichment in various influenza-related gene lists. The seven rankings consisted of network betweenness centrality, network degree centrality, differential gene expression (DE), combined betweenness and degree, combined betweenness and DE, combined degree and DE, and a combined ranking from all three. The average enrichment score in all influenza gene lists is shown for each of the seven rankings. Average enrichments were also calculated for 100 scrambled rankings of the same genes. P-values are calculated by comparing each ranking’s enrichment score to the distribution of enrichment scores of random rankings (see Methods). Single star indicates p-value below .05; double star indicates p-value below .001.

A recent study showed that combining the results of multiple inference algorithms produced a better-performing network than networks arising from individual algorithms [14]. We chose to follow this concept and combine topology metrics with DE expression to determine if merged rankings could confer better performance than individual rankings, based on a statistical measure of performance termed enrichment significance (Figure 4). We ranked the gene lists by the betweenness scores for bottlenecks, degree scores for hubs and the magnitude of difference for the DE genes. We also evaluated the combined rankings of any two of these three, and a combined ranking of all three metrics (see Methods). To evaluate the different influenza ranking metrics, we exploited the fact that several groups [23]–[29] have used interaction screens, knockdown analyses, and knowledge-based approaches to produce lists of host genes that are important for influenza virus infection. We used these lists to determine which of our influenza rankings, or combinations of them, showed enrichment in these previously-determined lists. To avoid the necessity of choosing arbitrary cutoffs for our ranked lists for enrichment analysis, we chose to use the Gene Set Enrichment Analysis (GSEA) tool, which identifies members of a collection of curated gene sets that show statistical enrichment near the top (or bottom) of a ranked gene list [30]. The statistical significance for each enrichment across the gene sets was incorporated into a single score (Methods), which was then compared to a series of enrichment analysis runs using gene lists with permuted rank order, representing background enrichment “noise” (Figure 5). Comparison of the rankings’ enrichment scores showed that the combined betweenness/degree ranking yielded the highest score, thus suggesting this ranking will also yield the highest proportion of novel regulators of influenza virus infection.

We note that the DE rank alone yields very low enrichment, which is likely responsible for the relatively low enrichment in the combined betweenness/degree/DE ranking. Figure S3 represents the significance level attained for each individual gene set in heat map form to demonstrate that the reference gene lists are complementary and not altogether overlapping. Interestingly, the list based on a comprehensive “expert” literature review attempting to identify the most relevant genes involved in host/influenza interaction (i.e. “Zhang et al” [29] in Figure S3) yielded high enrichment for all rankings, while lists based on experimental results (i.e. the other studies assessed in Figure S3) showed varying degrees of lesser enrichment.

**Figure 6:**
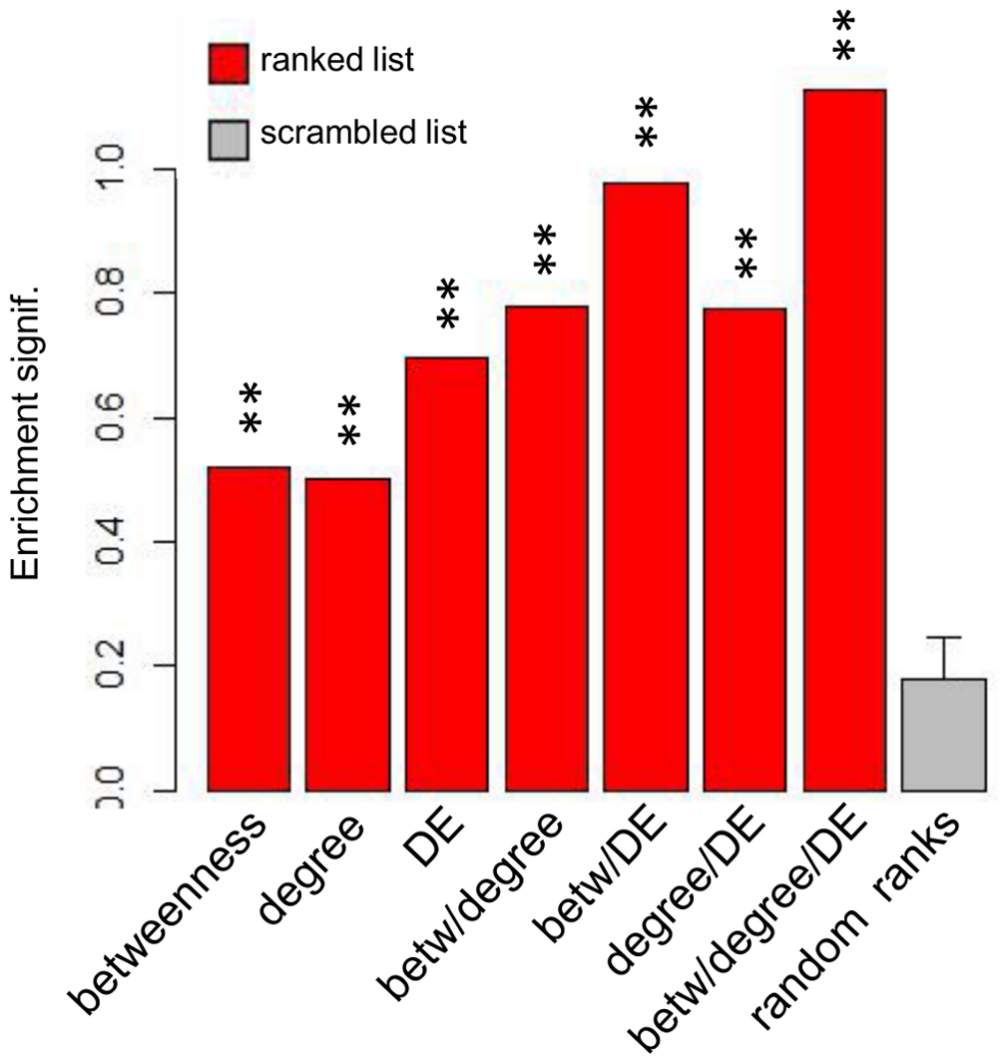
GSEA-based enrichment analysis of SARS-CoV rankings. Seven rankings of genes from the SARS-CoV network were assessed for enrichment as in figure 5, this time using the 299 gene sets from the Molecular Signatures Database matching the search keys “viral” or “virus”. Average scores are compared to random rankings. Double stars indicate p-values <0.001.

The approach described above allowed us to use current knowledge of influenza-related genes to predict the most ideal gene ranking for that virus. However, there is little comparable knowledge of SARS-CoV-related genes. While the influenza lists may be partly applicable to SARS-CoV, it is not known how reliable a comparison of this nature would prove. We therefore used gene sets available in msigdb, a large collection of over 8500 gene sets that represent pathways, cancer gene neighborhoods, genes downstream of various perturbations, transcription factor and miRNA target groups, chromosomal position, and gene ontology categories. Msigdb was searched for gene sets with names containing the terms ‘virus’ or ‘viral’, which yielded 299 sets. We reasoned that these gene sets would function well for enrichment analysis of general viral processes. We applied the virus-related enrichment analysis to the same SARS-CoV rankings from the integrated network in an identical manner as was applied to the influenza data (Figure 6). In contrast to the observations for influenza virus, the DE ranking showed strong enrichment for SARS-CoV, which conferred still higher enrichment on combined rankings that incorporated DE, with the combined degree/betweenness/DE ranking receiving the highest overall score (Figure 6). Comparing virus-related enrichment scores for SARS-CoV rankings and influenza-specific enrichment scores for influenza rankings showed that for both viruses, combining the hub and bottleneck ranking yielded better enrichment than for either ranking alone. A bootstrap approach (methods) showed this difference in enrichment to be statistically significant for SARS-CoV and influenza virus, and showed the degree/betweenness/DE ranking to be significantly higher than all other rankings for SARS-CoV. Figure S4 shows the enrichment of SARS rankings across the virus-related gene sets.

We utilized the respective highest-scoring rankings for SARS-CoV and influenza virus to predict regulatory candidates. The top 50 ranked genes resulting from these prioritizations are presented in Tables 2 and 3, with the complete rankings in Tables S1 and S2. To show the relationships in the network relevant to some of these genes, Figures S5–S10 display limited networks (connectivity to primary and secondary network neighbors) for the first three genes in each list with informative names. For SARS-CoV, these genes are CREB5, DUSP8, and NFKBIA (Figures S5, S6 and S7 respectively); for influenza virus they are PCGF5, NFE2L3 and HLA-E (Figures S8, S9 and S10 respectively).

**Table 2:** Top genes from SARS-CoV prioritization.

Symbol	entrez	refseq
CREB5	9586	NM_182898
DUSP8	1850	NM_004420
NFKBIA	4792	NM_020529
IL6	3569	NM_000600
TNFAIP3	7128	NM_006290
ZC3H12A	80149	NM_025079
ATF3	467	NM_004024
FAM46A	55603	NM_017633
NUAK2	81788	NM_030952
AXUD1	64651	NM_033027
GEM	2669	NM_005261
JUN	3725	NM_002228
NFKBIE	4794	NM_004556
ZNF433	163059	NM_001080411
HES1	3280	NM_005524
REL	5966	NM_002908
C3orf59	151963	NM_178496
BC018597	NA	BC018597
PTX3	5806	NM_002852
CH25H	9023	NM_003956
IL1A	3552	NM_000575
PPP1R15A	NA	NM_014330
TSC22D2	9819	NM_014779
X15675	NA	X15675
INHBA	3624	NM_002192
IL32	9235	NM_001012631
C3orf52	79669	NM_024616
NPFFR2	10886	NM_053036
DNAJA4	55466	NM_018602
HSPA6	3310	NM_002155
LOC442229	NA	BC024198
ENST00000342294	6434	ENST00000342294
ZBTB10	65986	NM_023929
MAP3K14	9020	NM_003954
CCRN4L	25819	NM_012118
IKZF3	22806	NM_012481
M74509	NA	M74509
RELB	5971	NM_006509
LOC401317	9586	ENST00000381802
TMEM16C	63982	NM_031418
BF514513	27	BF514513
IL29	282618	NM_172140
PIM3	415116	NM_001001852
NR1D1	9572	NM_021724
CXCL2	2920	NM_002089
CCNYL1	NA	NM_152523
PER1	5187	NM_002616
TNF	7124	NM_000594
OVOL1	5017	NM_004561
EYA1	2138	NM_000503

**Table 3:** Top genes from influenza regulatory prioritization.

gene.symbol	entrez	refseq
LOC652411	NA	XR_019314
PCGF5	84333	BC007377
NFE2L3	9603	NM_004289
CA314451	NA	CA314451
HLA-E	3133	NM_005516
LOC646626	NA	XM_942822
SEMA7A	8482	NM_003612
BC089454	NA	BC089454
AK056449	NA	AK056449
TNF	7124	NM_000594
AK026497	7528	AK026497
LRP4	4038	NM_002334
THC2670384	NA	THC2670384
NLRP3	NA	NM_004895
HLA-C	NA	BC002463
SALL2	6297	NM_005407
PML	5371	NM_002675
SPAG5	10615	NM_006461
HBEGF	1839	NM_001945
CIT	11113	NM_007174
THC2621771	NA	THC2621771
NT5E	4907	NM_002526
SEMA3A	10371	NM_006080
ENST00000342294	6434	ENST00000342294
DDX58	23586	NM_014314
KIAA1704	55425	AB051491
ID1	3397	NM_002165
UBL3	5412	NM_007106
AK129584	NA	AK129584
PLK3	NA	NM_004073
BC033829	9590	BC033829
BC064492	7329	BC064492
CCDC6	8030	NM_005436
FAM83E	NA	NM_017708
HCP5	NA	L06175
CRSP2	9282	NM_004229
CD69	969	NM_001781
HOXB6	3216	NM_018952
SLC16A2	6567	NM_006517
MICAL2	9645	NM_014632
CPT1C	126129	NM_152359
DB340110	NA	DB340110
C20orf142	128486	BC029662
SORT1	6272	NM_002959
SPATA1	1.01E+08	NM_001081472
HLA-B	3106	NM_005514
ADRB2	154	NM_000024
A_23_P66347	NA	A_23_P66347
ND1	NA	ENST00000361390
KIAA1370	56204	NM_019600

After establishing the GSEA enrichment ranking approach, we used it to test whether the conservative proteome/transcriptome integration approach described above conferred an improvement over transcriptome networks alone. Betweenness centrality of networks with and without proteome data integration was evaluated. SARS-CoV networks showed a significant enrichment increase in betweenness ranking when proteome data were incorporated (p = 0.0029). No improvement was seen with integration of influenza proteome data, but this is not surprising given that no dramatic shifts in bottleneck position were observed in our analysis above. To determine the effect of incorporating random edges (using the conservative approach) into the SARS-CoV transcriptome network, an equivalent number of random edges were added to the network and the resulting new betweenness was calculated. Rather than improving the enrichment as seen when the proteome edges were added, random edge integration caused less enrichment, although the difference did not reach significance (p = 0.46). Although the vertices in the integrated SARS-CoV network consist of both transcripts and proteins, for convenience we will use the term “genes” to refer to all network vertices from this point forward.

### Building a Predictive Model to Assess Conservation

**Figure 7:**
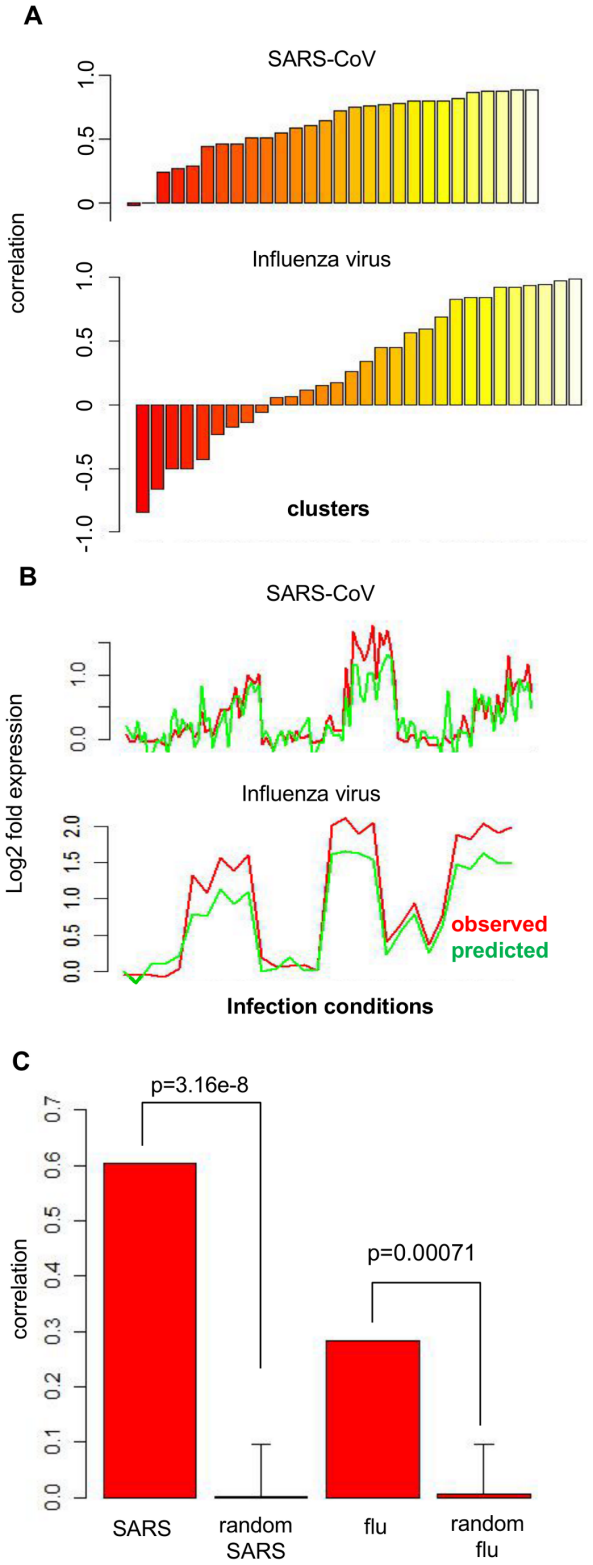
Model system comparison based on Inferelator regression models. Regulatory influence models for each gene cluster of both viruses were applied to comparable datasets from distinct model systems. For SARS-CoV, regulatory influences inferred from Calu3 data were applied to SARS-CoV infection data from a primary human airway epithelial cell model system. For influenza, the Calu3 model was applied to influenza infection data from C57BL/6 mice. The observed gene expression profile of the non-Calu3 data clusters was compared to the predicted gene expression profile based on the Calu3 model. Correlations were calculated for this comparison from each cluster and are shown in A. In B, a sample expression profile from a highly-predictive cluster from each virus is shown with the observed non-Calu3 expression profile shown in red, compared to the predicted expression profile from the Calu3 model in green. In C, the average cluster correlation for the SARS-CoV and influenza comparisons is shown, in comparison to the correlation obtained from applying 100 random models to the corresponding alternative model system. P-values were obtained by comparing each correlation with the distribution of 100 correlations based on random models.

A critical question regarding the use of model systems is how well the experimental model represents the target system. Calu3 cells provide a convenient *in vitro* model for lung epithelium, but it is unknown to what extent the pathways and mechanisms found in the *in vitro* model will translate to a more realistic model, such as primary cells or *in vivo* animal models. In order to demonstrate that our approach based on *in vitro* data provides predictive insights relevant to an *in vivo* model, we used the Inferelator software [31] to determine how well models derived from Calu3 data would apply to data from other experimental systems [18] (steps 3 and 4 in Figure 2). The Inferelator uses multivariate regression to select a small set of regulators that are the most likely to be influencing a regulatory target. Expression data from the Calu-3 cells were collapsed using hierarchical clustering, such that clusters of genes with similar expression profiles were chosen as regulatory “targets”. A list of candidate regulators was supplied to the Inferelator software as input, which was compiled from known transcription factors and the top candidate regulators from our ranking strategy [32]. A prediction of the critical regulators of a gene cluster can be used to predict the behavior of that gene cluster based on the behavior of its predicted regulators. Behavioral conservation can therefore be evaluated by comparing predicted cluster expression profiles with expression data in a distinct, but comparable biological system. We applied the SARS-CoV model to a dataset of SARS-CoV infection of primary human airway epithelial (HAE) cells. These cultures are an *in vitro* model of the human lung that morphologically and physiologically recapitulates the epithelium of the conducting airway and have been demonstrated to be permissive for SARS-CoV infection [33]. The cultures differentiate from primary cells into ciliated, goblet (mucin-producing), and basement membrane cells in a stratified epithelium. For influenza data, the Calu3 model was fitted to a set of mouse influenza infection experiments using VN1203 to infect mice with 10^3^, 10^4^, or 10^5^ PFU at 1 and 2 days post-infection [18]. Calu3 models were applied to new data (HAE for SARS-CoV, mouse for influenza virus) by using the expression levels of the input regulators (measured in the new system) to calculate the expression level of each gene cluster in the new system, using the model learned from the Calu3 experimental system. Figures 7A and B show the correlation of Calu3 models’ predicted output of each gene cluster with the observed output of the actual *in vivo* or *ex vivo* data. Since the SARS-CoV Calu3/HAE comparison is within the human system and within a cell culture context, while the influenza Calu3/mouse comparison spans distinct species *and* model system types, these comparisons from the two virus types yielded somewhat different results. Unsurprisingly, overall, the Calu3-based model of SARS-CoV infection was more compatible with HAE data than was the Calu3 model of influenza infection with the mouse data. However, assessing agreement for individual clusters showed that 6 out of 28 clusters yielded correlation above 0.8 in SARS-CoV, while 9 out of 30 clusters had correlation above this level in influenza virus. As an illustration, the expression profile of a single high performing cluster prediction from each virus is included for comparison with the observed expression of the cluster (Figure 7b). The high performing SARS-CoV clusters were enriched for genes involved in the immune response. The two highest performing influenza clusters were also enriched for immune response genes, while another showed enrichment for transcription regulation. For each virus type, we also generated 100 models from datasets with scrambled genes but with the cluster structure intact. In this way, we compared the observed level of cross-model fitting with the level of model fitting expected by random chance. Figure 7c shows the average Pearson’s correlation of the clusters’ predicted profiles and the observed profiles from the new data. The average correlation of models derived from data with scrambled rows is shown for comparison.

While these results demonstrate the high degree of regulatory conservation between the different models, we decided to identify the individual genes that displayed the most conserved response to infection across model systems. To this end, we used the cross-prediction metric (Xpred) described in [18], which is a measure of how well the behavior of a gene is predicted across model systems. Xpred rankings are shown in Table S3 (SARS-CoV) and Table S4 (influenza virus). To determine if these genes showed enrichment in functional categories, we used GO term enrichment analysis on genes with the highest Xpred scores in each of the two virus datasets. For both viruses, genes with a conserved response showed functional enrichment in innate immune response signaling (not shown). Thus while not all regulatory mechanisms are conserved between model systems, a component of the innate immune response is well-preserved.

We next wished to determine the overlap between these genes showing conservation of behavior between model systems, and the genes predicted previously from regulatory candidate ranking (tables 2 and 3). This intersection represents genes from each virus dataset that are both behaviorally conserved across different systems, and are predicted to fill regulatory roles. Figure 2 illustrates the process of isolating these genes. Intersection of the top 5% of each of the two rankings yielded highly significant overlaps, with 37 overlapping genes for SARS (p-value∼2e-6, genes in table 4) and 24 for influenza (p-value = 0.00043, genes in table 5). We refer to genes highly ranked in both categories as “conserved regulators”.

**Table 4:** Conserved regulators from SARS-CoV virus.

Gene symbol	entrez	Refseq
CREB5	9586	NM_182898
DUSP8	1850	NM_004420
NFKBIA	4792	NM_020529
IL6	3569	NM_000600
**TNFAIP3**	**7128**	**NM_006290**
ZC3H12A	80149	NM_025079
ATF3	467	NM_004024
AXUD1	64651	NM_033027
JUN	3725	NM_002228
BC018597	NA	BC018597
PTX3	5806	NM_002852
IL1A	3552	NM_000575
HSPA6	3310	NM_002155
ZBTB10	65986	NM_023929
CCRN4L	25819	NM_012118
LOC401317	9586	ENST00000381802
IL29	282618	NM_172140
CXCL2	2920	NM_002089
PER1	5187	NM_002616
**TNF**	**7124**	**NM_000594**
PMAIP1	5366	NM_021127
IL28A	282616	NM_172138
SOCS1	8651	NM_003745
HDAC9	NA	NM_058176
IFNB1	3456	NM_002176
CXCL10	3627	NM_001565
CCL20	6364	NM_004591
CD274	29126	ENST00000381577
C1orf38	NA	BC031655
ZC3HAV1	56829	NM_024625
HSPB8	NA	NM_014365
NCOA7	135112	NM_181782
THSD7A	221981	ENST00000262042
TRAF1	7185	NM_005658
FLJ25801	205860	NM_173553
IL28B	NA	NM_172139
MT1B	4490	NM_005947

Bold: genes overlapping with the influenza virus list.

**Table 5:** Conserved regulators from influenza virus.

Gene symbol	entrez	Refseq
**TNF**	**7124**	**NM_000594**
LRP4	4038	NM_002334
HBEGF	1839	NM_001945
DDX58	23586	NM_014314
CD69	969	NM_001781
SLC16A2	6567	NM_006517
YRDC	79693	NM_024640
**TNFAIP3**	**7128**	**NM_006290**
RGS16	6004	NM_002928
ZNFX1	57169	NM_021035
PAK1IP1	55003	NM_017906
SDCBP	6386	NM_005625
L3MBTL2	83746	NM_031488
MPHOSPH10	10199	NM_005791
TAP1	6890	NM_000593
ARL3	403	NM_004311
NMI	9111	NM_004688
PARP12	64761	NM_022750
IL4I1	259307	NM_172374
STAG3	10734	NM_012447
SAA2	NA	NM_030754
CBX7	23492	NM_175709
EIF4A2	NA	NM_001967
ISG20	3669	NM_002201

Bold: genes overlapping with the SARS-CoV list.

Interestingly, the overlap of conserved regulators *across* viruses consisted of two genes: TNF and TNFAIP3, which are both obvious regulators of the inflammatory response. The fact that both regulators common to both viruses are potentially involved in inflammation underscores the critical role this function plays in the pathogenicity of respiratory viruses.

## Discussion

The intent of this study was to use in vitro models of severe respiratory viral infection to predict key elements of the host response. We used a novel integrated network ranking approach to identify promising candidates for future studies that are predicted to play critical roles in viral infection. We implemented a four-stage analysis workflow to achieve this goal, with each stage involving comparison with and incorporation of several sources of outside data. Corroboration with independent studies both greatly strengthened and validated our approach, thus increasing the value of the candidates we propose.

We inferred separate transcriptome and proteome association networks as a foundation for our analysis due to the inherent differences in their data structure and dynamic range. To verify that the inference procedure produced valid edges, predicted network edges were compared with pairings of transcription factors with predicted targets based on promoter sequences. Because of the size of the transcriptome network, a large number of regulatory interactions were expected by chance. However, the inferred networks contained significantly more “known” edges than expected by chance. We also corroborated our network construction in a completely separate manner by comparing neighbors of vertices in our networks to genes affected by siRNA knockdowns in a distinct system and again found much greater overlap than would be expected by chance.

Despite the limited nature of proteome data, protein expression levels supply important information that transcriptome data cannot provide. Due to regulation at translational, post-translational and protein stability levels, mRNA levels are not strictly correlated to protein expression and sometimes are not correlated at all. Previous studies demonstrated that integration of proteome data into transcriptome networks improved network performance [7], [11], [34]. For the current study, we demonstrated an improvement in network performance when SARS-CoV proteome and transcriptome data were integrated. No corresponding improvement was observed from integration of influenza proteome data, which may be related to the fact that unlike the SARS-CoV data sets, not all experimental conditions present in the influenza transcriptome data set had corresponding proteome data. Further, the slower kinetics of SARS-CoV compared to influenza virus may affect what can and can’t be observed as protein abundance changes before cell death occurs. The fact that SARS-CoV doesn’t kill cells for several days after infection may allow a better chance for recovery of proteome data influence.

In the past, various methods have been employed to predict “important” vertices from network topology analysis. Multiple network centrality measures have been shown to identify high centrality vertices that are of greater interest than vertices with low centrality. To predict key regulators of respiratory virus infection, we chose to focus on betweenness and degree centrality, since these have been shown to be of interest in the study of inferred networks derived from high-throughput biological data [2], [4]–[6], [20]–[22]. In addition to network topology, genes/proteins that are highly DE are of potential interest. Employing the “wisdom of crowds” concept [14], we hypothesized that some/all of these prioritization strategies could be combined to produce an optimized ranking, such that the most influential/critical genes are ranked the highest. Enrichment analysis from both SARS-CoV and influenza showed that combinations of multiple rankings resulted in higher enrichment than individual rankings alone. Hubs and bottlenecks have previously been separately shown to represent important regulatory components of biological systems, however we show that for both SARS-CoV and influenza virus, a combination of these two network centrality measures yielded a higher-performing ranking than either ranking alone (Figures 5–6). This finding suggests that genes that are (to some extent) hubs *and* bottlenecks are most relevant to SARS-CoV and influenza infection. For influenza, this combined ranking received the highest enrichment, while a combination of degree centrality, betweenness centrality and DE was the highest overall performing ranking for SARS-CoV. Interestingly, the influenza DE ranking performed very poorly, far below topological measures and comparable to randomly ordered rankings. This was in contrast to the SARS-CoV DE ranking which compared even better than individual topology-based rankings. While the reason for poor influenza DE performance is unknown, it may be related to our observation that 96% of all genes in the influenza compendium exhibited more extreme differential expression in HP than LP strains, while only 61% of genes examined in the SARS-CoV analysis showed this trend. The overwhelming surge in gene expressing seen in HP influenza virus may thus have obscured any enrichment by flooding the analysis with significantly changed genes. Despite the absence of a DE component in the influenza virus regulatory prioritization, the dominant expression pattern of HP influenza likely exerts a strong influence in the network topology, so that topological measures capture features of HP virus.

Another aspect of analysis validation that is frequently ignored is the question of how accurately one model system represents the target system. While complete ‘omics studies of human respiratory infections are not available, we did have access to data from comparable experiments that could be thought of as more representative of human infection, which allowed us to determine the comparability of different model systems. For SARS-CoV, we compared Calu3 infection to a well-differentiated, primary cell epithelial model, in which physiological features such as active cilia, mucus production and an air-liquid interface are present [33], [35]. Using regression to identify the most likely regulators of clusters in the data, we showed that the selected set of regulatory influences for each gene cluster in the Calu3 model was able to predict the behavior in the differentiated model relatively well, with a correlation as high as 0.89, with most clusters showing correlation above 0.5. Interestingly, despite the obvious differences between *in vitro* cell culture systems and animals, the comparison of Calu3 influenza infection to flu-infected mice showed correlation coefficients greater than 0.50 for 12/30 clusters (Figure 7a). The fact that the behavior of several clusters was successfully predicted in the mouse suggests that some aspects of infection are preserved across systems. We observed that the most accurately predicted gene clusters from both SARS-CoV and influenza virus showed functional enrichment for pathways related to the immune response (data not shown); thus innate immunity may be one aspect of infection that is well-preserved across multiple models of infection. We used the regression results to identify individual genes whose regulation displayed high conservation between systems, then combined this ranking with the results of our regulatory prediction process to predict conserved regulators.

Due to the known relevance of the specific genes isolated by our analysis to severe viral infection, the results of our analysis indicate a successful outcome. This is demonstrated by the presence of genes such as TNF and DDX58 in the influenza conserved regulators, which are known to be important regulators during influenza virus infection [36]–[38], and suggests that our prioritization approach has successfully promoted important genes to the top of the rankings. Similarly, DUSP8, IL6, CXCL10, and NFKBIA are up-regulated in SARS patients [39], [40], while PTX3 [41] and CXCL2 [42], [43] have been shown to be involved in SARS-CoV infection. Further, the top 100 genes in the SARS-CoV list of regulators includes all three JNK/p38-specific MAPK phosphatases (DUSP8, DUSP10, and DUSP16), which is significant since SARS-CoV is known to up-regulate JNK [44], [45]. All three DUSP genes are highly up-regulated (data not shown), perhaps representing a negative feedback loop as the host cell attempts to counteract the virus-induced JNK activation. Interestingly, the predicted conserved regulators for SARS-CoV identified the CREB/ATF family members CREB5 and ATF3 as highly ranked. We determined that the best ranking method for the SARS-CoV dataset was a combined DE/hub/bottleneck metric, such that designation as a conserved regulator for SARS-CoV required relatively high differential expression between strains of differing pathogenicity, high network degree ranking, and high network betweenness, in addition to exhibiting conserved behavior between Calu3 and the primary airway epithelial model. CREB5 was ranked #1 in the combined SARS-CoV ranking (out of 7186), while ATF3 was ranked #8. CREB5 was not at all highly ranked in influenza network topology measures, however its differential expression profile exhibited down-regulation in HP virus, and up-regulation in LP strains (not shown). CREB/ATF family transcription factors are known to integrate signaling from a wide array of pathways, resulting in both gene activation and repression. Little is known about the specific pathways regulated by CREB5, however ATF3 is known to mediate repression of inflammatory signals, and may function as an oncogene or tumor suppressor depending on cell type and context [46]. While CREB5 and ATF3 have a generally strong induction pattern in SARS-CoV, the induction is relatively mild for influenza virus. The intra-and inter-virus comparison of the expression patterns of these genes, as well as their placement in the topology of the SARS-CoV network suggest that signaling through one or both these molecules is an important component of SARS-CoV pathogenesis that is distinct from influenza virus. The overlap between the influenza virus and SARS-CoV conserved regulators appears to highlight the importance of the inflammatory response in both viruses. The role of TNF and TNFAIP3 in inflammation is well known, and TNFAIP3 has been shown previously to play a specific role in influenza infection [47].

The development and evaluation of these methods involved comparing to, or integrating with other sources of experimental data, thus providing a measure of validation (Figure 2 indicates steps where outside data was integrated). For the first stage, edges in the inferred networks were compared with relationships acquired from separate sources (predicted transcription factor binding sites and experimentally observed siRNA targeting effects) to show statistically significant agreement. For the second stage, ranking method selection was based entirely on how well the ranking demonstrated enrichment in experimentally-derived gene sets. For the last stage, the final selection of conserved regulators was performed by identifying regulators with similar behavior in distinct, highly relevant model systems, thus providing a final level of validation from independent studies.

In conclusion, we have used a systems biology approach to predict a subset of genes/proteins likely to function as key regulators of SARS-CoV and influenza, respectively, using integrated transcriptome and proteome data. We have used crowd-based approaches and multivariate regression to prioritize the most likely candidates, and have introduced a novel approach to metric selection using the GSEA software. The resulting high-ranking genes provide a rich set of research directions for ours and other groups interested in respiratory viral infections to pursue in the future.

## Materials and Methods

### Ethics Statement

Human tracheobronchial epithelial cells were obtained from previously de-identified airway specimens resected from patients undergoing surgery under University of North Carolina Institutional Review Board-approved protocols by the Cystic Fibrosis Center Tissue Culture Core.

### Infections

For influenza virus, Calu3 infections and sample preparation for transcriptomics of VN1203 and NL602 are described in detail in [17]. Data from these experiments was published previously [17]. CA04 infection and sample collections were performed exactly as described for NL602. Briefly, Calu3 cells were infected with VN1203 at multiplicity of infection (MOI) of 1 for 0, 3, 7, 12, 18, and 24 h or CA04 or NL602 at MOI of 3 for 0, 3, 7, 12, 18, 24, 30, 36, and 48 h.

For SARS-CoV, infections were performed in either Calu3 2B4 cells, a clonal population of Calu3 cells (human lung adenocarcinoma) sorted for high levels of expression of the SARS-CoV cellular receptor, angiotensin converting enzyme 2 (ACE2) [48], or in human primary tracheobronchial epithelial cell cultures. Calu3 2B4 cells were grown in minimal essential media (MEM) (Invitrogen-Gibco) containing 20% fetal bovine serum (Hyclone) and 1% antibiotic anti-mycotic (Invitrogen-Gibco). Viral titration assays were performed in VeroE6 cells. VeroE6 cells were maintained in MEM (Invitrogen-Gibco) containing 10% Fetal Clone II (Hyclone) and 1% antibiotic anti-mycotic (Invitrogen-Gibco).

Human airway epithelium cultures (HAE) were generated by provision of an air-liquid interface for 6 to 8 weeks to form well-differentiated, polarized cultures that resemble *in vivo* pseudo-stratified mucociliary epithelium [35].

Wild type infectious clone derived SARS-CoV (icSARS-CoV), icSARS-CoV ΔORF6 and Bat-SRBD were derived from the Baric laboratory’s infectious clone constructs as previously described [12], [15], [16]. Briefly, genome fragments were amplified in E. coli, ligated, and purified prior to in vitro transcription reactions to synthesize full length genomic RNA which were transfected into VeroE6 cells. All work was performed in a BSL3 facility supported by redundant fans. Research staff wore tyvek suits, gloves, aprons and booties and portable air breathing apparatus (PAPR) as specified by the manufacturer (3M).

For infections of Calu3 2B4 cells, the cells were plated in triplicate for each condition at each time point, washed prior to infection, infected with MOI of 5 (meaning 5 infectious virus particles per cell) for icSARS-CoV ΔORF6 or 1 for Bat-SRBD (each with wild type icSARS-CoV at the specified MOI) and incubated at 37°C for 40 minutes. The inoculum was then removed, cells were washed 3 times with 1XPBS, and then fresh media added prior to time 0. For both microarray and proteomics analysis, at 0, 3, 7, 12, 24, 30, 36, 48, 54, 60, and 72 hours post infection, media was collected to determine viral titers at each time point for each well and cells were either washed in 1XPBS, and then harvested in TRIzol (Invitrogen) and stored at -80°C (RNA) or washed 3 times in cold 150 mM ammonium bicarbonate buffer, lysed for 15 minutes in 8M urea and stored at −80C (protein). Infection of HAE cultures with icSARS-CoV, icSARS-CoV ΔORF6, and Bat-SRBD was performed as previously described [33], [49], [50]. Briefly, triplicate cultures were washed with 1XPBS and 200uL of mock, icSARS-CoV, icSARS-CoV ΔORF6 or Bat-SRBD inoculum (MOI 2) added to the apical surface. Cultures were incubated at 37°C for 2 hours, the inoculum removed and unbound viruses removed by washing three times with 1X PBS. Apical wash samples were harvested to analyze viral growth kinetics at 0, 12, 24, 36, 48, 60, 72, and 96 hours post infection and were assayed by plaque assay in Vero E6 cells [33]. Total RNA was harvested by washing the apical and basolateral surfaces of the cultures with 1XPBS and then adding 500uL of TRIzol to the apical surface, incubating for 5 minutes and transferring to a fresh tube. Samples were then frozen at −80C until being sent for processing.

### Proteomics

Detailed proteomics methodology, including sample preparation, processing and analysis, are provided in Supporting Information S1. Calu3 cells lysates were trypsin digested and fractionated by strong cation exchange (SCX) as previously described [51], [52]. A novel accurate mass and time (AMT) tag database [53] was generated for each sample type by LC-MS/MS analysis [51], [54] of each SCX fraction and LC-MS analyses were subsequently performed on each individual unfractionated sample to generate quantitative data using identical chromatographic and electrospray conditions as for LC-MS/MS analyses. For quantitative analyses, the LC system was interfaced to an Exactive mass spectrometer (Thermo Scientific), and the temperature of the heated capillary and the ESI voltage were 250°C and 2.2 kV, respectively. Data were collected over the mass range 400–2,000 *m/z*. Quantitative LC-MS datasets were processed using the PRISM Data Analysis system [55], which is a series of software tools developed in-house (e.g. Decon2LS [56] and VIPER [57] freely available at http://ncrr.pnl.gov/software/). Individual steps in this data processing approach are reviewed here [53]. The peak intensity values (i.e. abundances) for the final peptide identifications were processed in a series of steps using MatLab® R2010b, including quality control [58], normalization [59], and quantification to protein level [60]. Comparative statistical analyses of time-matched mock samples with infected samples per sample type were performed using a Dunnett adjusted t-test to assess differences in protein average abundance, and a G-test to assess associations among factors due to the presence/absence of response [61].

### Microarrays

RNA isolation, array hybridization, signal processing, normalization and QC filtering was performed as described in [17]. Briefly, RNA was isolated from infected cells, quantified, and hybridized to Agilent 4×44K human HG arrays. Raw data extracted from image analysis were background corrected and normalized with quantile normalization.

### Compendia

We made compendia of all data for each virus type, such that data from all HP and LP strains were included. Data were included for each probe where at least one time point demonstrated differential expression with respect to time-matched mocks, using the criteria of a minimum fold change of 2.0 and maximum FDR-corrected p-value of 0.05. Compendia were assembled for transcriptome and proteome datasets. To deal with missing values in proteome data, a conservative approach was adapted, in which the missing values of a given experimental condition were filled in with the average value for the replicates present for that condition, only if at least half of the replicates were present in that condition. Proteins for which one or more conditions showed more than half missing values were discarded.

### Network Inference

Compendia were used as input to the Context Likelihood of Relatedness (CLR) software [3], which uses assessment of mutual information to generate a matrix representing the pairwise relationships between all genes. CLR runs were set with parameters: bins = 10; spline = 3. To determine an appropriate CLR cutoff value for incorporation of a gene vertex into the inferred network, we generated influenza networks using multiple cutoffs, calculated betweenness for all vertices in each network, and used several functional gene lists [23]–[29] to test the top-scoring genes for functional enrichment. A CLR threshold of 2.0 was chosen to generate all subsequent networks.

### Edge Validation

Transcription factor/target pairs were taken from msigdb subgroup C3:TFT, downloaded from http://www.broadinstitute.org/gsea/msigdb/collections.jsp. All network edges were compared to the database edges to determine overlap, similar to [3]. To determine the background levels of accidental edge inference, one of the parent vertex columns in each network edge file was scrambled, thus generating a random network based on the same number of vertices and edges. These new edges were then compared with the database edges, and the permutation process was repeated 1000 times. P-values were calculated from z-scores derived from the size of database overlap, and the mean and standard deviation of the overlaps of random networks with the database.

Based on a dataset of 400 different transcriptome profiles of HUVEC having been knocked down by siRNA transfection [19] available on the NCBI-GEO database via the ascension number GSE27871, we identified the list of significant differentially expressed genes (fold-change≥1.5, p<0.05) compare to a control condition (“TNFa untreated” condition) for each knocked-down condition. Moreover, for each gene identified in the influenza or SARS inferred co-expression networks for which a knocked-down profile was available, we identified the list of adjacent genes in the inferred network. Then a p-value representing the statistical significance of the overlap between the differentially expressed genes identified based on the knocked down profiles and the network neighbors was calculated using the right-tailed Fisher’s exact test. P-values under.01 were counted as significant. For both the influenza and SARS-CoV networks, we generated 500 random networks having the same topological structure by randomly permuting the vertices but by keeping the edges fixed. For each generated random network, we performed the same overlap test with the HUVEC dataset as before. Final p-values (in figure 3B) were obtained from z-scores derived by comparing the actual percentage of genes that showed significant overlap with the distribution of percentages obtained using random networks.

### Differential Expression Ranking

To establish a ranking for genes highly differentially expressed between HP and LP, we used the difference between the expression levels of HP and LP at each time point. (Log2 experimental:mock ratios were used for initial compendia construction.) For the SARS-CoV data, we took the ΔORF6 mutant and the bat SRBD strain as LP for comparison with HP wild type SARS-CoV. We used ANOVA and the Tukey’s test to determine which of the differences between HP and LP strains were significant. The absolute value of the difference at each significant time point was summed for each gene yielding a DE score:
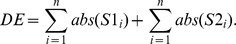
where S1 is the set of differences between HP and LP strains at time points where the difference was statistically significant in ΔORF6, and S2 is the similar set of values for bat SRBD. For influenza, we used the VN1203 data as HP, and the Ca04 and NL602 strains as LP. In contrast to the SARS-CoV experiments which were performed with wild type virus alongside mutants within each experiment, influenza strains were all used in separate experiments, thus making determination of fold change significance between strains problematic. We therefore simply took the sum of the absolute value of the HP and LP differences at all time points for each gene.

With this approach, the DE behavior of each gene is collapsed into a single value for all temporal stages of the infection process. Since a large number of genes show a response to influenza virus infection in the first 12 hours, we only included these time points in the DE ranking to avoid incorporation of secondary regulatory effects in our analysis. This time point-specific ranking was not performed for the SARS-CoV data, since SARS-CoV infected cells do not show the same early response, and no obvious demarcation between early and late gene expression is evident.

### Combined Rankings

We combined topological rankings and DE scores for each gene into a single prioritization score. This was done by converting each score into a ranking based on its position in a sorted list of rankings for all genes, then using the equivalent quantiles to find an average:
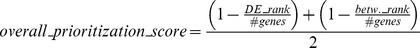
A similar procedure was followed to combine three rankings into one composite ranking.

### Network Integration

Proteome vertices of edge file entries (from CLR-derived proteome networks, see above) were converted to probe IDs using the biomaRt package in R. For complete integration, converted proteome edge entries were simply added to transcriptome edge entries. These new combined edge files were used to build integrated networks; all vertices in the networks were then assessed for betweenness and degree centrality. For conservative integration, converted proteome edges were identified for which both parent vertices existed in the transcriptome network, but no edge between them existed. These edges were then combined with the transcriptome edges and all other proteome edges were discarded.

### GSEA-based Ranking Assessment

The “GSEA preranked” setting in the GSEA software was used to determine enrichment of gene sets at the top and bottom of a preranked list of genes [30]. Either the custom set of 7 influenza-related gene sets, or the set of msigdb gene set names with a match to the words “viral” or “virus” was used as the reference gene set collection. The FDR corrected enrichment significance values were converted to −1* log10, and these values were averaged over all gene sets, resulted in an overall enrichment score. Enrichment scores were compared to 100 enrichment scores of scrambled rankings of the same genes to obtain p-values using the z-test. P-values were calculated from z-scores derived from the enrichment score, and the mean and standard deviation of 100 enrichment scores of scrambled rankings.

### Bootstrap GSEA Ranking Calculation

A bootstrap approach was used to identify significant differences between individual GSEA enrichment scores resulting from the above method. Each ranked list of genes was resampled with replacement; this new ranking was equivalent to the original but contained a random subset of the original genes with their respective rankings. The new ranking was then assessed for GSEA-based enrichment as described above. The distribution of enrichment scores derived from 100 iterations of this process was compared to distributions from other rankings derived in the same way. Standard ANOVA and post-hoc tests were used to determine which, if any, of the enrichment scores of the original rankings were statistically different from each other.

### Inferelator-based Modeling

The Inferelator software (May 2008, version 1.2) was used to infer critical regulatory influences in our datasets. Required input to the program includes sets of candidate regulators and regulatory targets. Genes known to function as transcription factors (all mouse genes with the GO annotation “transcription factor activity”; acquired from http://www.informatics.jax.org/) were provided as regulators to the Inferelator software [32]. To ensure that a rich selection of candidate regulators was available for modeling, the top 500 ranked genes from the betweenness/degree/DE combined ranking were chosen as additional SARS-CoV regulators, and the top 500 ranked genes from the betweenness/degree were chosen as additional influenza virus regulators. For regulatory targets, merged proteome/transcriptome network from both viruses were used to derive adjacency matrices, which were subsequently used for hierarchical clustering using Ward’s minimum variance method [62]. The average expression profiles of these clusters were used as the regulatory targets. Number of clusters was determined by running the Inferelator with cluster levels from 5 to 50, and the cluster level at which the predictive model could best replicate the observed data was chosen as the optimal cluster level (28 clusters for SARS-CoV, 30 for influenza virus). Inferelator was run with the max.inter.corr.cutoff parameter set to −1, so as to prevent calculation of the effect of dimeric regulators.

### Model System Comparison

Inferelator models derived from SARS-CoV and influenza virus-infected Calu3 datasets were compared to the observed expression levels in SARS-CoV–infected HAE cells and influenza virus-infected mice, respectively as described in [18]. To summarize, the cluster expression levels in the HAE and mouse systems were predicted using the assigned regulatory weights of the Inferelator models from Calu3 and the regulator expression levels from the non-Calu3 data. To improve the comparability of the mouse dataset, only days 1 and 2 post-infection were used in the comparison, despite data from days 4 and 7 being also present. In this way we determined the applicability of regulatory influences in Calu3 to other systems. Individual genes were ranked by their cross-prediction (Xpred) score [18] to prioritize genes with high correlations in both the relationship between the predicted regulatory mechanisms in the Calu3 model and the target model (HAE or mouse), and between each gene and its parent clusters overall behavior.

### Data Dissemination

Raw microarray data have been deposited in NCBI’s Gene Expression Omnibus [63] and are accessible through Gene Expression Omnibus (GEO) SuperSeries accession GSE47963 (http://www.ncbi.nlm.nih.gov/geo/query/acc.cgi?acc=GSE47963), as well as SubSeries accessions GSE47960 (http://www.ncbi.nlm.nih.gov/geo/query/acc.cgi?acc=GSE47960), GSE47961 (http://www.ncbi.nlm.nih.gov/geo/query/acc.cgi?acc=GSE47961), and GSE47962 (http://www.ncbi.nlm.nih.gov/geo/query/acc.cgi?acc=GSE47962). Raw proteomics data corresponding to peptide identifications used to populate the AMT tag database are available at the PRoteomics IDEntification (PRIDE) database (http://www.ebi.ac.uk/pride/) under the project name A Systems Biology Approach to Emerging Respiratory Viral Diseases in the PRIDE Public Projects folder and corresponding to PRIDE Accession numbers 19878 (H5N1) 19877–19890. The raw quantitative proteomics data can be accessed at the PNNL Biological MS Data and Software Distribution Center (http://omics.pnl.gov/) in the Systems Virology Contract Data folder within the Browse Available Data folder. All data sets and associated metadata have been submitted to Virus Pathogen Resource (ViPR, http://www.viprbrc.org). Additional details from this study and similar studies can be accessed through the Systems Virology website (http://www.systemsvirology.org). If these data are used in additional publications please acknowledge the Systems Virology Center, NIAID Contract No. HHSN272200800060C.

## Supporting Information

Figure S1 **Placement of proteome vertices in ranked betweenness lists.** All vertices in the integrated network were ordered according to betweenness score, and vertices originating from proteome data were identified. Placement of proteome vertices in the betweenness ranking was indicated using a histogram. Top panels represent complete incorporation of proteome vertices, while bottom panels represent integration using the conservative approach (see text and Figure S2).
(TIF)

Figure S2 **Schematic illustrating conservative integration of proteome edges into transcriptome network.** To avoid spurious network structure, only proteome edges are merged into the transcriptome network for which both parent vertices are already present in the transcriptome network. This causes changes in the network structure altering the betweenness score for some genes (depicted at left).
(TIF)

Figure S3 **Individual enrichment scores for each of the 7 gene sets used to evaluate influenza rankings.** Colors indicate the individual enrichment scores for each influenza gene ranking with each influenza-related gene list.
(TIF)

Figure S4 **Individual enrichment scores for general gene sets used to evaluate SARS-CoV rankings.** Colors indicate enrichment scores of each SARS-CoV ranking for 299 gene sets from diverse categories obtained from msigdb. Gene set sub-categories are indicated on the right.
(TIF)

Figure S5 **Limited CLR network of connections to CREB5, #1 on the list of predicted regulators for SARS-CoV (** **Table 2** **).** Targeted node is colored red, primary neighbors are colored dark pink, secondary neighbors are colored light pink.
(JPG)

Figure S6 **Limited CLR network of connections to DUSP8, #2 on the list of predicted regulators for SARS-CoV (** **Table 2** **).** Nodes are colored as in Figure S5.
(JPG)

Figure S7 **Limited CLR network of connections to NFKBIA, #3 on the list of predicted regulators for SARS-CoV (** **Table 2** **).** Nodes are colored as in Figure S5.
(JPG)

Figure S8 **Limited CLR network of connections to PCGF5, #2 on the list of predicted regulators for** Influenza **virus (** **Table 3** **).** Nodes are colored as in Figure S5.
(JPG)

Figure S9 **Limited CLR network of connections to NFE2L3, #3 on the list of predicted regulators for** Influenza **virus (** **Table 3** **).** Nodes are colored as in Figure S5.
(JPG)

Figure S10 **Limited** CLR **network of connections to HLA-E, #5 on the list of predicted regulators for Influenza virus (** **Table 3** **).** Nodes are colored as in Figure S5.
(JPG)

Supporting Information S1 (DOC)

Table S1 **Combined SARS-CoV ranking.** Ranking of genes for the SARS-CoV analysis based on the combined ranking of betweenness, degree centrality and differential expression between pathogenicity levels. Corresponds to step #2 in Figure 2. High ranked genes are referred to as candidate regulators. Columns contain various identifiers as indicated.
(ZIP)

Table S2 **Combined influenza virus ranking.** Ranking of genes for the influenza virus analysis based on the combined ranking of betweenness and degree centrality. Corresponds to step #2 in Figure 2. High ranked genes are referred to as candidate regulators. Columns contain various identifiers as indicated.
(ZIP)

Table S3 **Conservation ranking for SARS-CoV.** Conservation ranking based on agreement of regulatory model inferred in SARS-CoV infected Calu3 with data from SARS-CoV infection of human primary airway epithelium. Xpred is defined in [18]. Remaining columns contain various identifiers as indicated.
(ZIP)

Table S4 **Conservation ranking for influenza virus.** Conservation ranking based on agreement of regulatory model inferred in influenza virus infected Calu3 with data from influenza virus infection of mice. Xpred is defined in [18]. Remaining columns contain various identifiers as indicated.
(ZIP)
