# Supplementary figures and images for: A Network Integration Approach to Predict Conserved Regulators Related to Pathogenicity of Influenza and SARS-CoV Respiratory Viruses
Source: PLoS One. 2013 Jul 25;8(7):e69374. doi: 10.1371/journal.pone.0069374 (PMC3723910; doi:10.1371/journal.pone.0069374)

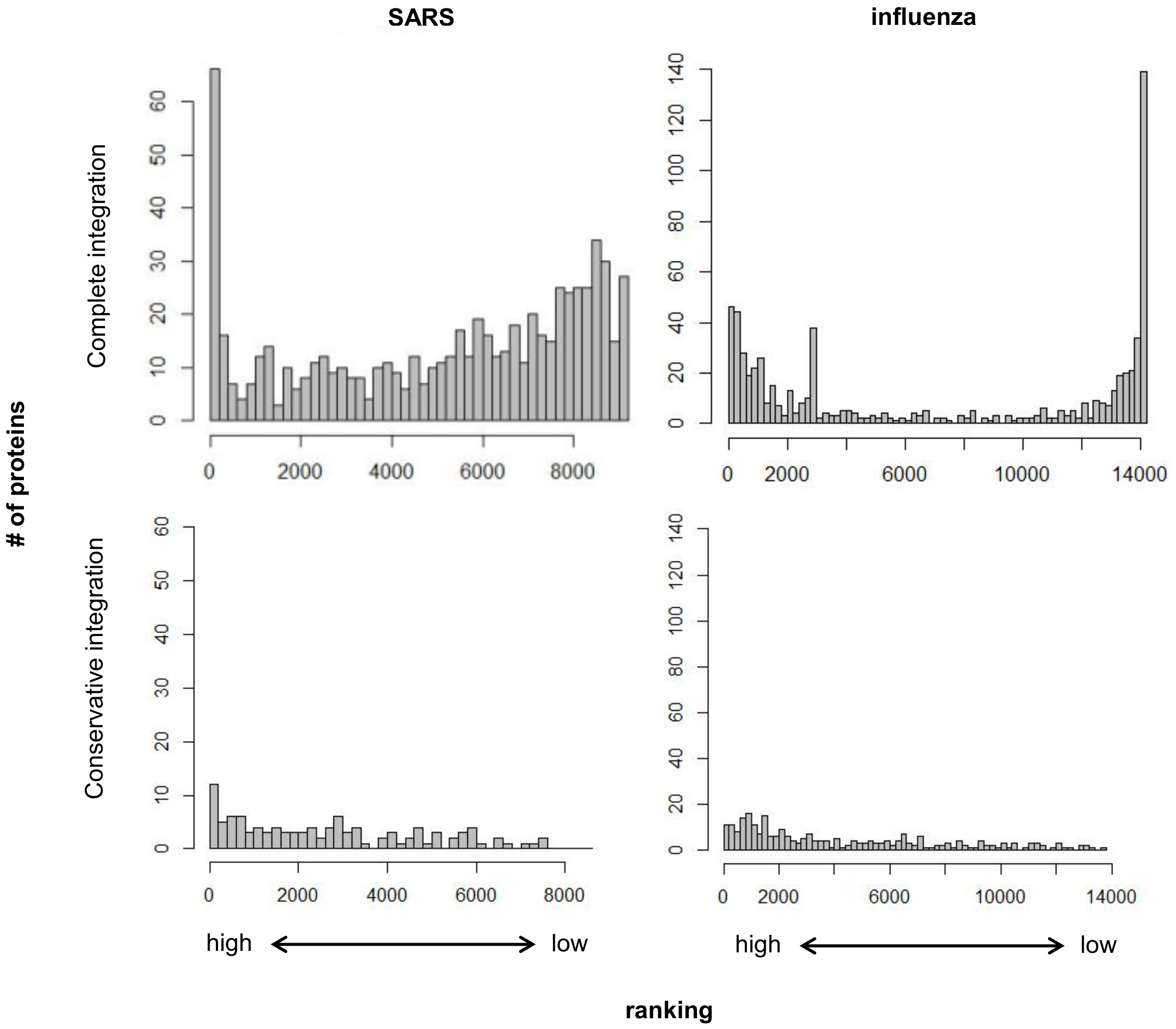

Supplement: Figure S1 — Placement of proteome vertices in ranked betweenness lists. All vertices in the integrated network were ordered according to betweenness score, and vertices originating from proteome data were identified. Placement of proteome vertices in the betweenness ranking was indicated using a histogram. Top panels represent complete incorporation of proteome vertices, while bottom panels represent integration using the conservative approach (see text and Figure S2). (TIF) [file pone.0069374.s001.tif]

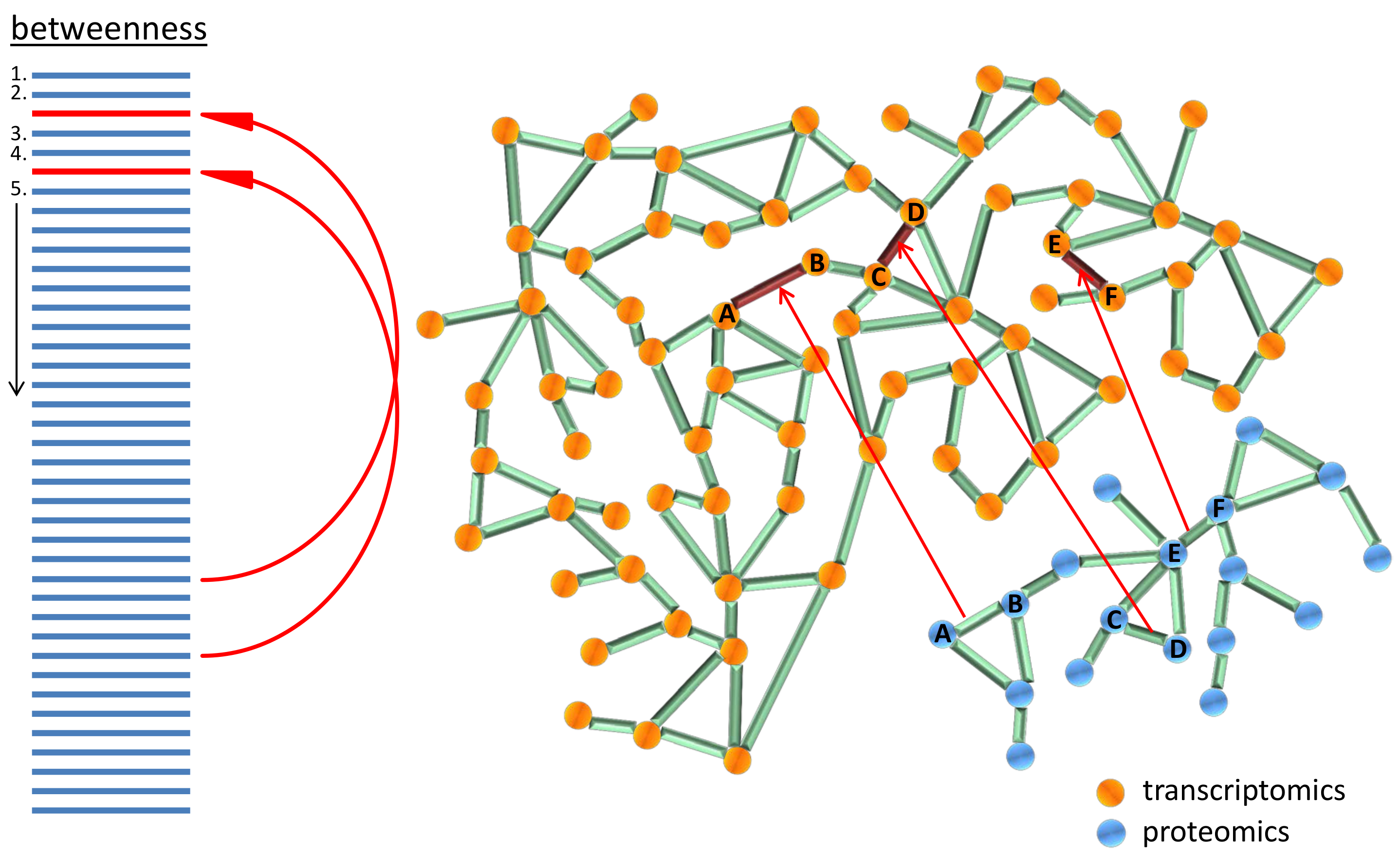

Supplement: Figure S2 — Schematic illustrating conservative integration of proteome edges into transcriptome network. To avoid spurious network structure, only proteome edges are merged into the transcriptome network for which both parent vertices are already present in the transcriptome network. This causes changes in the network structure altering the betweenness score for some genes (depicted at left). (TIF) [file pone.0069374.s002.tif]

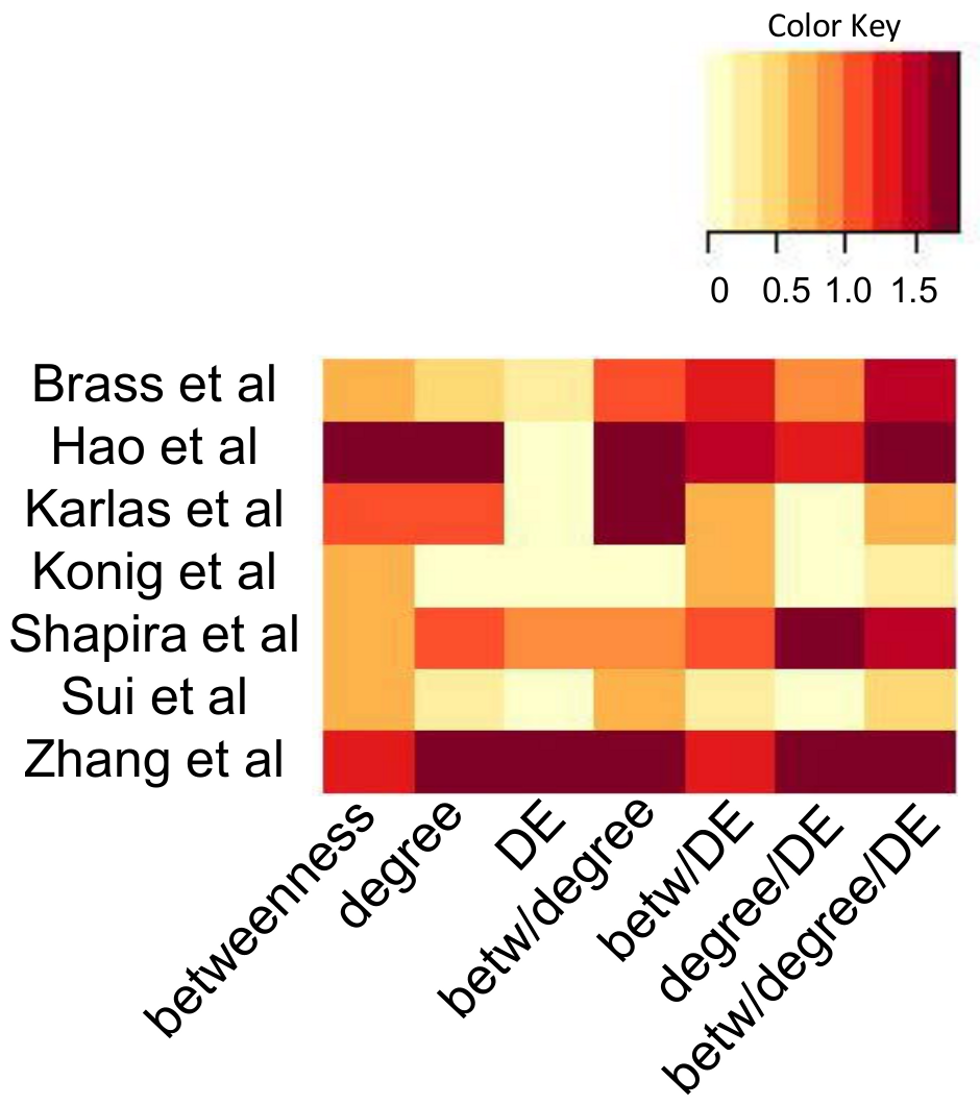

Supplement: Figure S3 — Individual enrichment scores for each of the 7 gene sets used to evaluate influenza rankings. Colors indicate the individual enrichment scores for each influenza gene ranking with each influenza-related gene list. (TIF) [file pone.0069374.s003.tif]

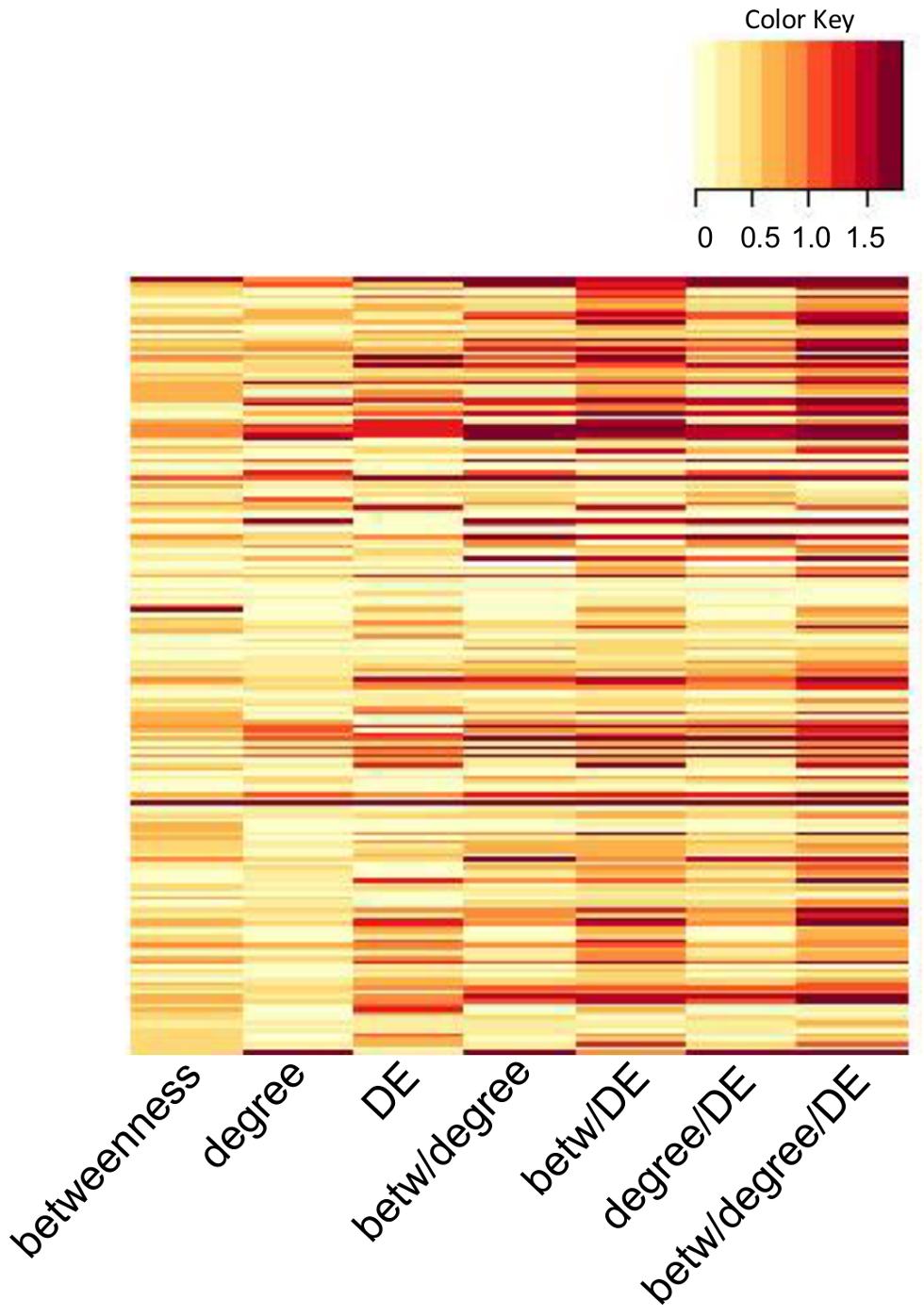

Supplement: Figure S4 — Individual enrichment scores for general gene sets used to evaluate SARS-CoV rankings. Colors indicate enrichment scores of each SARS-CoV ranking for 299 gene sets from diverse categories obtained from msigdb. Gene set sub-categories are indicated on the right. (TIF) [file pone.0069374.s004.tif]

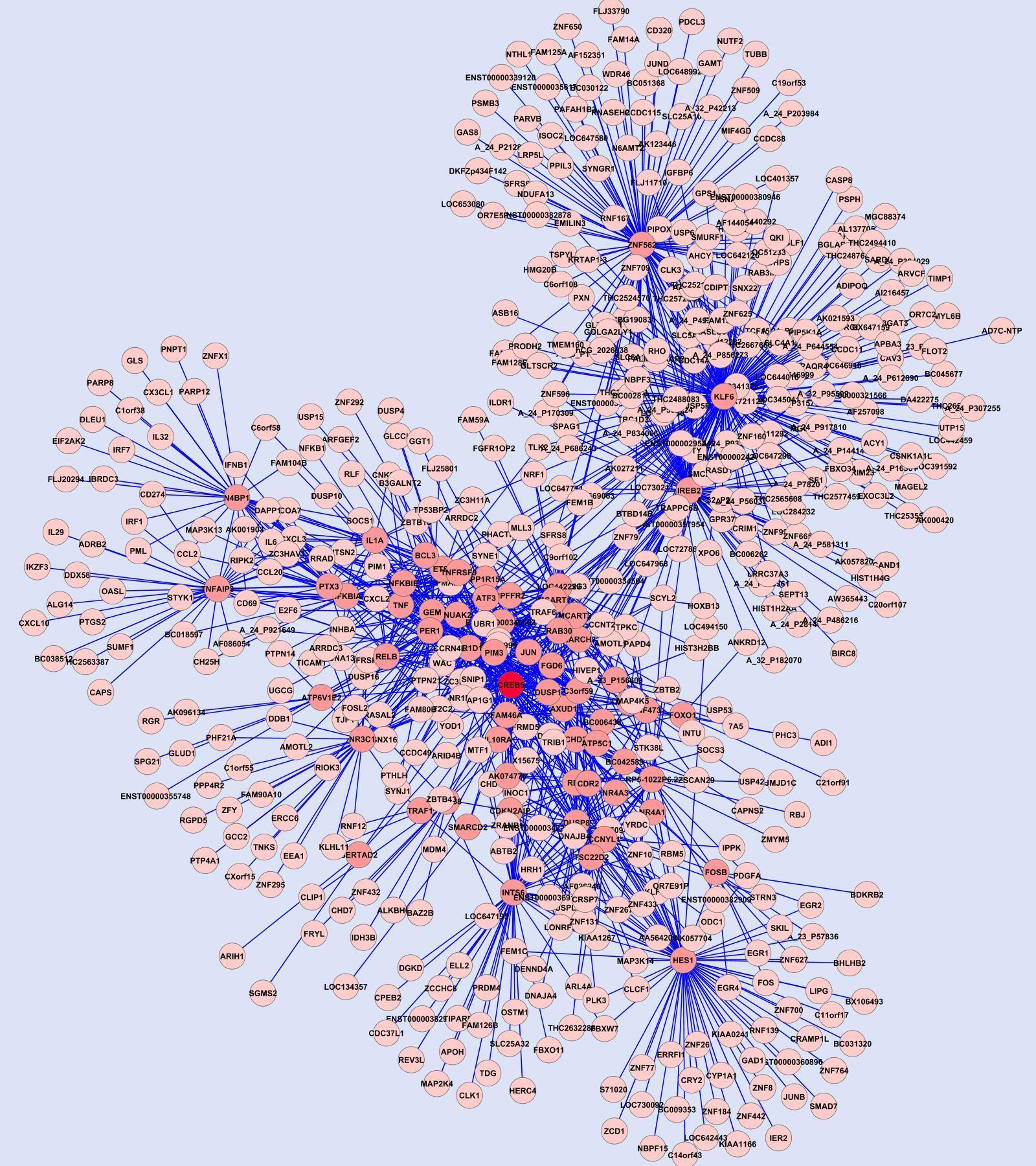

Supplement: Figure S5 — Limited CLR network of connections to CREB5, #1 on the list of predicted regulators for SARS-CoV ( Table 2 ). Targeted node is colored red, primary neighbors are colored dark pink, secondary neighbors are colored light pink. (JPG) [file pone.0069374.s005.jpg]

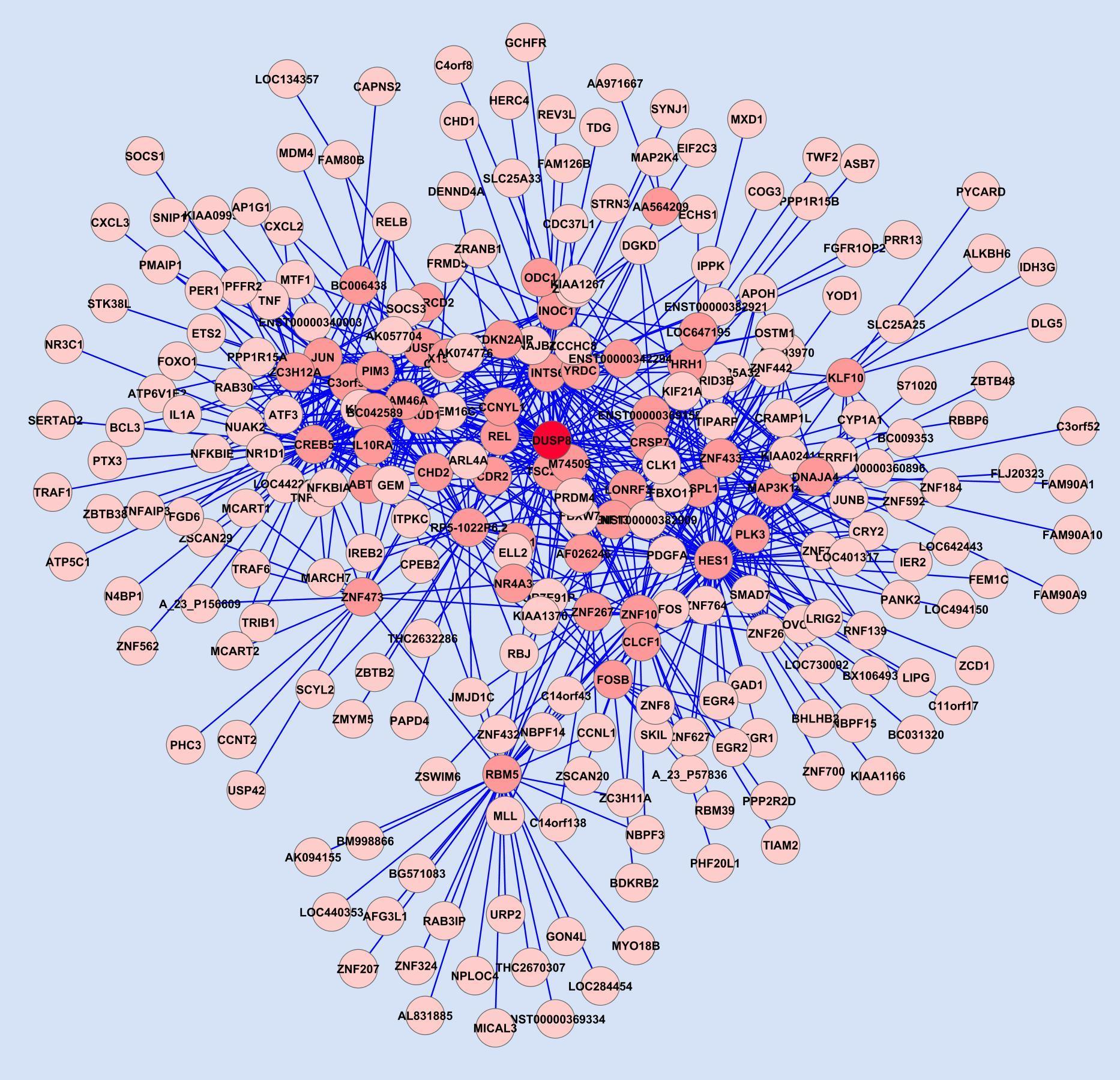

Supplement: Figure S6 — Limited CLR network of connections to DUSP8, #2 on the list of predicted regulators for SARS-CoV ( Table 2 ). Nodes are colored as in Figure S5. (JPG) [file pone.0069374.s006.jpg]

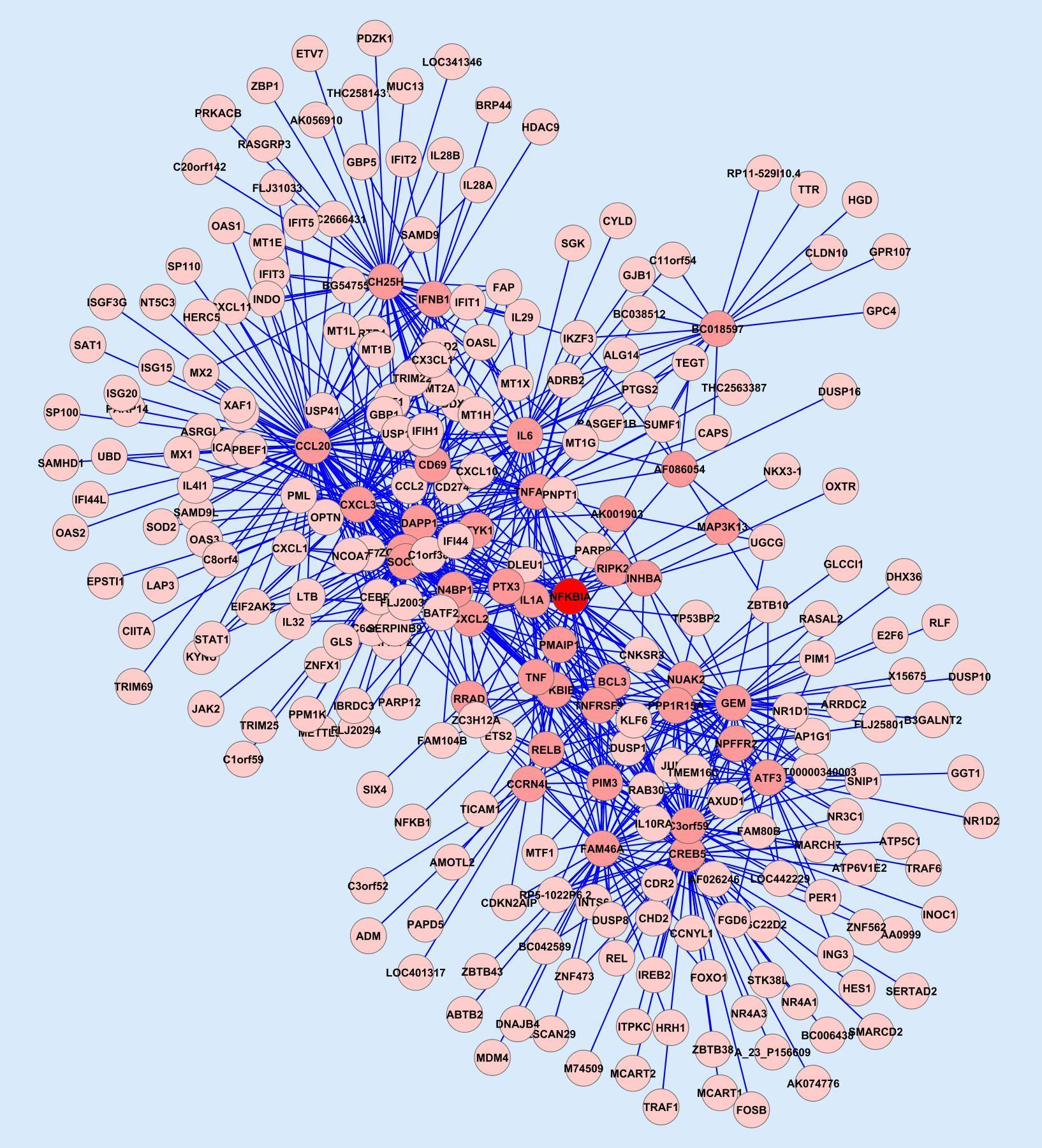

Supplement: Figure S7 — Limited CLR network of connections to NFKBIA, #3 on the list of predicted regulators for SARS-CoV ( Table 2 ). Nodes are colored as in Figure S5. (JPG) [file pone.0069374.s007.jpg]

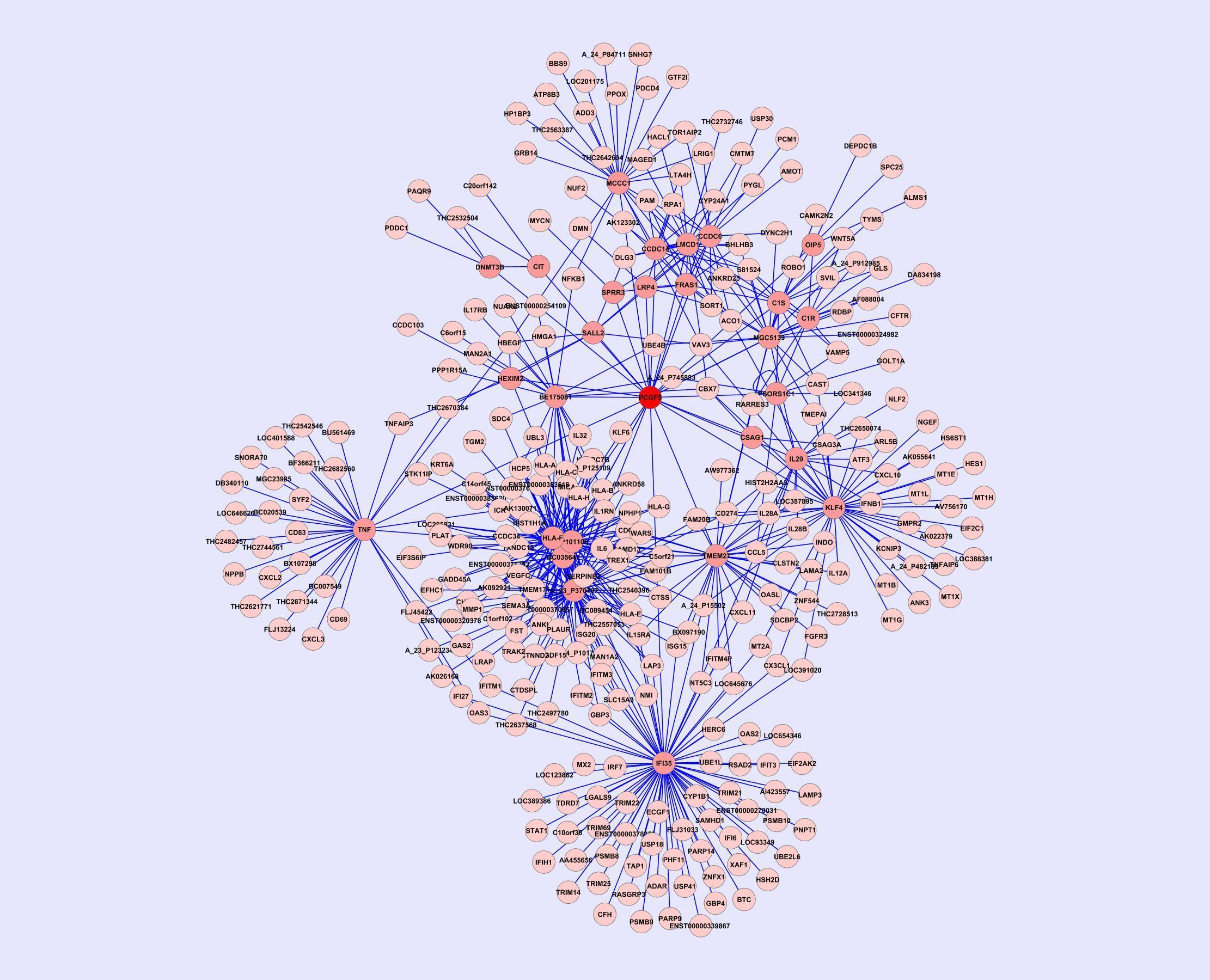

Supplement: Figure S8 — Limited CLR network of connections to PCGF5, #2 on the list of predicted regulators for Influenza virus ( Table 3 ). Nodes are colored as in Figure S5. (JPG) [file pone.0069374.s008.jpg]

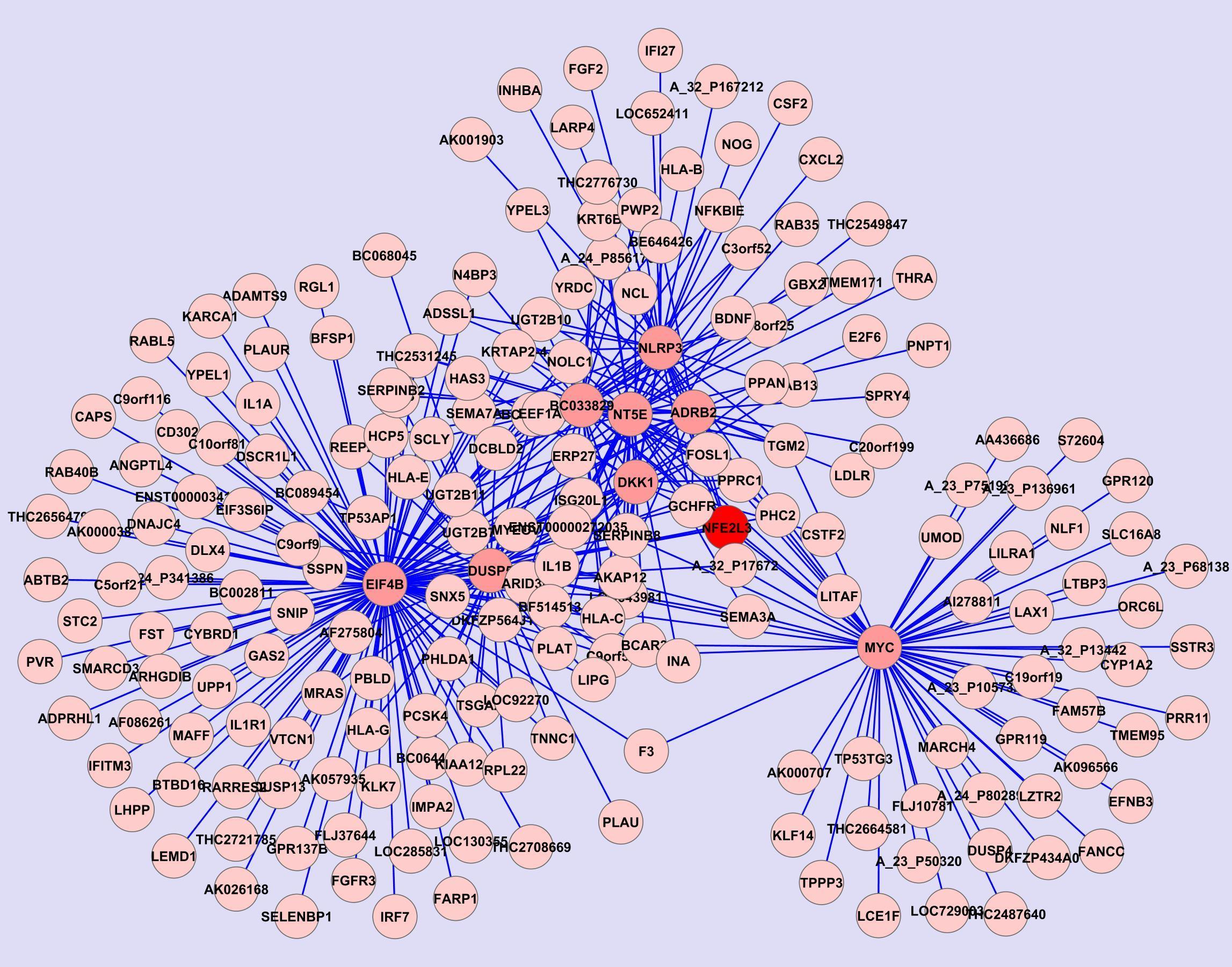

Supplement: Figure S9 — Limited CLR network of connections to NFE2L3, #3 on the list of predicted regulators for Influenza virus ( Table 3 ). Nodes are colored as in Figure S5. (JPG) [file pone.0069374.s009.jpg]

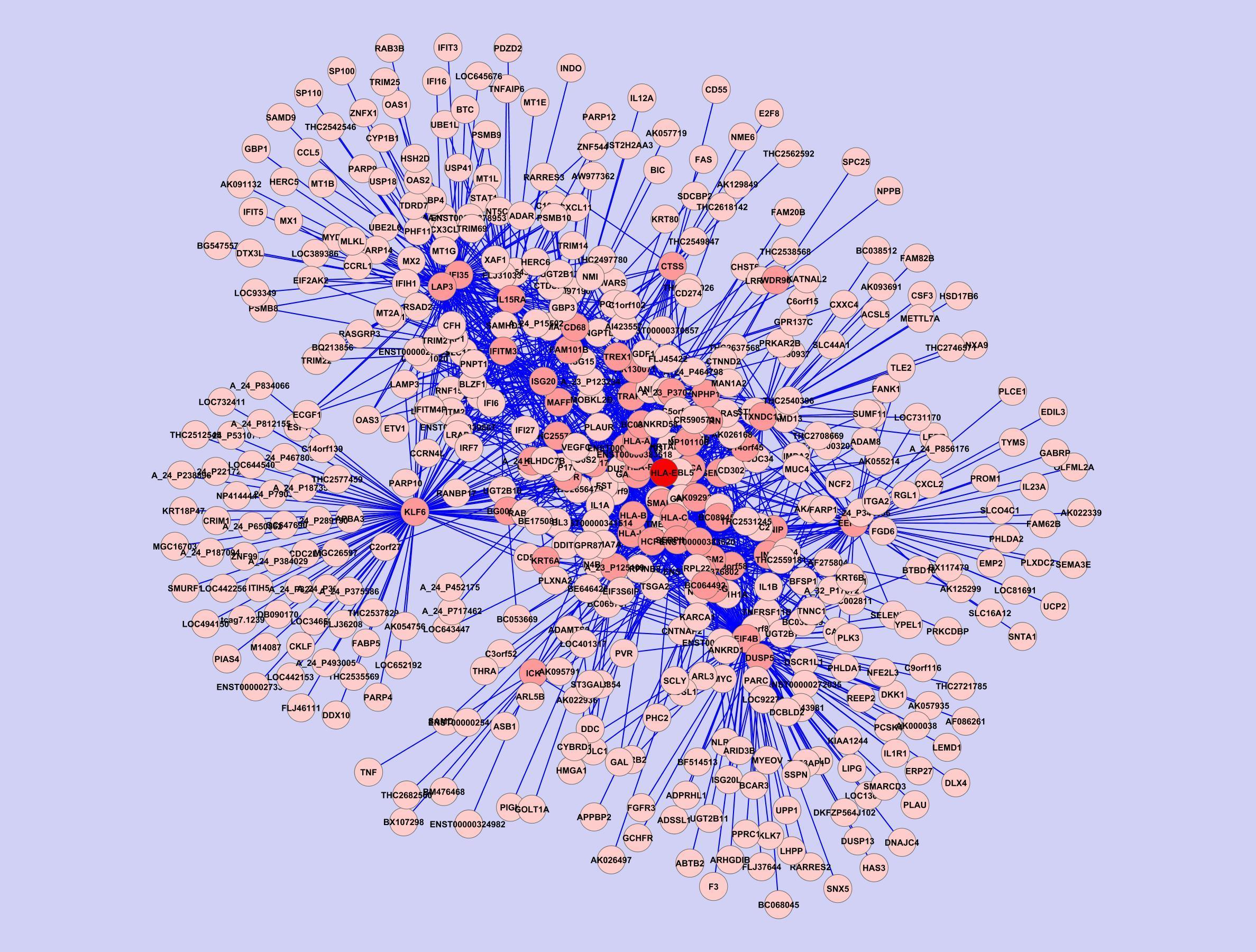

Supplement: Figure S10 — Limited CLR network of connections to HLA-E, #5 on the list of predicted regulators for Influenza virus ( Table 3 ). Nodes are colored as in Figure S5. (JPG) [file pone.0069374.s010.jpg]
