# Supplementary material for: A Network Integration Approach to Predict Conserved Regulators Related to Pathogenicity of Influenza and SARS-CoV Respiratory Viruses
Source: PLoS One. 2013 Jul 25;8(7):e69374. doi: 10.1371/journal.pone.0069374 (PMC3723910; doi:10.1371/journal.pone.0069374)
Supplement: Supporting Information S1 — (DOC) [file pone.0069374.s011.doc]

**Supporting Information S1: Proteomic Methods**

**Proteomics reagents and sample preparation and strong cation exchange fractionation.** All chemicals and reagents were purchased from Sigma-Aldrich (St. Louis, MO) unless stated otherwise. Ammonium bicarbonate and acetonitrile were purchased from Fisher Scientific (Pittsburgh, PA), and sequencing-grade modified trypsin was purchased from Promega (Madison, WI). Bicinchoninic acid (BCA) assay reagents and standards were obtained from Pierce (Rockford, IL); purified, deionized water, >18 MΩ, (Nanopure Infinity ultrapure water system, Barnstead, Dubuque, IA) was used to make all aqueous buffers.

Cell lysate and lung homogenate protein concentrations were determined by BCA protein assay and diluted to a uniform final volume in 50 mM ammonium bicarbonate pH 7.8. Proteins were reduced with 10 mM dithiothreitol, followed by alkylation of free sulfhydryl groups with 40 mM iodoacetamide in the dark; each reaction was performed for 1 h at 37°C with constant shaking at 800 rpm. Denatured and reduced samples were diluted 10-fold with 50 mM ammonium bicarbonate pH 7.8, and CaCl_2_ was added to a final concentration of 1 mM prior to enzymatic digestion. Sequencing-grade modified trypsin was activated by adding 20 μL of 50 mM ammonium bicarbonate pH 7.8 to 20 μg lyophilized trypsin and incubating for 10 min at 37˚C. Activated trypsin was then added to the samples at 1:50 (w/w) trypsin-to-protein ratio, and samples were digested at 37˚C for 3 h with constant shaking at 800 rpm; reactions were quenched by rapid freezing in liquid nitrogen. Digested samples were desalted using solid phase extraction columns (Discovery C18, Supelco, Bellefonte, PA) according to the manufacturer’s instructions. Samples were then concentrated to 100 μL *in vacuo* (Speed-Vac SC 250 Express, Thermo Savant, Holbrook, NY), and a BCA protein assay was performed to verify final peptide concentrations. Samples were stored at –80˚C until strong cation exchange fractionation with liquid chromatography-tandem mass spectrometry (LC-MS/MS) or quantitative LC-MS analyses. Strong cation exchange fractionation was performed as previously described . Twenty-four fractions were collected from minute 30 to minute 65 of the gradient, and they were subsequently dried *in vacuo* and stored at -80˚C until LC-MS/MS analysis.

**Reversed-phase capillary LC-MS/MS and LC-MS analyses.** LC-MS/MS analysis was used to generate an accurate mass and time (AMT) tag database for both virus-infected Calu3 2B4 cells and virus-infected mouse lung homogenates (see below). For this, dried peptide fractions were reconstituted in 30 µL of 25 mM ammonium bicarbonate pH 7.8 and analyzed using a 4-column custom-built capillary LC system coupled online to a linear ion trap mass spectrometer (LTQ; Thermo Scientific, San Jose, CA) by way of an in-house manufactured electrospray ionization interface, as previously described . To identify the eluting peptides, the LTQ was operated in a data-dependent MS/MS mode as previously described .

Following Calu3 2B4/virus AMT tag database generation, LC-MS analyses were performed on all individual unfractionated infected and mock-infected samples to generate quantitative data. For this, dried peptide samples were reconstituted in 30 µL of 25 mM ammonium bicarbonate, pH 7.8 and analyzed in triplicate and random order using identical chromatographic and electrospray conditions as for LC-MS/MS analyses. The LC system was interfaced to an Exactive mass spectrometer (Thermo Scientific), and the temperature of the heated capillary and the ESI voltage were 250°C and 2.2 kV, respectively. Data were collected over the mass range 400-2,000 *m/z*.

**Development of the AMT tag database for virus-infected Calu-3 cells.** A novel AMT database was generated for Calu-3 cells using mock-infected and virus infected cells. To generate the AMT tag database, aliquots of the samples were combined to make the following pools per respective virus infection: 1) mock-infection (all time points; mocks from SARS and influenza experiments were pooled separately), 2) early infection (0, 12, 24, and 36 h for SARS; 0, 3, and 7 h for influenza), and 3) late infection (48, 54, 60, and 72 h for SARS; 12, 18, and 24 h for influenza). Similarly, an AMT tag database was generated for mouse lung using mock-infected and virus infected homogenates. Again, aliquots of the samples were combined to make the following pools per respective virus infection: 1) mock-infection (all time points; mocks from SARS and influenza experiments were pooled separately), 2) early infection (1 and 2 d; both viruses), and 3) late infection (4 and 7 day; both viruses). Each pool was subjected to fractionation, and each fraction was analyzed by LC-MS/MS. The SEQUEST analysis software was used to match the MS/MS fragmentation spectra with sequences from the April 4, 2010 UniProt/Swiss-Prot protein database, containing 20,276 entries (protein TITIN_Human was removed due to excessive length). When searching, SEQUEST used a dynamic mass modification on methionine residues corresponding to oxidation (15.9949 Da) and a static mass modification on cysteinyl residues to account for alkylation by iodoacetamide (57.0215 Da). Cell peptides passing the following filter criteria were stored as AMT tags in a Microsoft SQL Server database: 1) SEQUEST DelCn2 value (normalized Xcorr difference between the top scoring peptide and the second highest scoring peptide in each MS/MS spectrum) ≥ 0.10 and 2) SEQUEST correlation score (Xcorr) ≥ 2, 2.6, and 3.5 for fully tryptic peptides with 1+, 2+, and 3+ charge states, respectively, and Xcorr ≥ 2.5, 3.6, and 4.1 for partially tryptic or non-tryptic protein terminal peptides with 1+, 2+, and 3+ charge states, respectively. Similarly, lung peptides passing the following filter were stored as AMT tags: 1) SEQUEST DelCn2 value (normalized Xcorr difference between the top scoring peptide and the second highest scoring peptide in each MS/MS spectrum) ≥ 0.10 and 2) SEQUEST correlation score (Xcorr) ≥ 1.6, 2.4, and 3.2 for fully tryptic peptides with 1+, 2+, and 3+ charge states, respectively, and Xcorr ≥ 4.3 and 4.7 for partially tryptic or non-tryptic protein terminal peptides with 2+ and 3+ charge states, respectively. Non-tryptic peptides were excluded, and a minimum peptide length of 6 amino acid residues was required for both cell and lung datasets. These criteria resulted in 56,220 cell and 39,744 lung peptides identified with an estimated false discovery rate <2% based on a target-decoy database search . The elution times for these peptides were normalized to a range of 0 to 1 using a predictive peptide LC normalized elution time (NET) model and linear regression, as previously reported . A NET average and standard deviation were assigned to each identified peptide if the same peptide was observed in multiple analyses. Both calculated monoisotopic masses and observed NETs of identified peptides were included in the AMT tag database.

**Processing of quantitative LC-MS datasets.** Quantitative LC-MS datasets were processed using the PRISM Data Analysis system , which is a series of software tools developed in-house (e.g. Decon2LS and VIPER freely available at http://ncrr.pnl.gov/software/). Individual steps in this data processing approach are reviewed here . The peptide identities of detected features in each dataset (here a dataset is equivalent to a single LC-MS analysis) were determined by comparing their measured monoisotopic masses and NETs to the calculated monoisotopic masses and observed NETs of each of the peptides in the filtered AMT tag databases within initial search tolerances of ± 6 ppm and ± 0.025 NET for monoisotopic mass and elution time, respectively. The peptides identified from this matching process were retained as a matrix for subsequent data analysis.

**Proteomic data processing**. The peak intensity values (i.e. abundances) for the final peptide identifications were processed in a series of steps using MatLab® R2010b.  Briefly, quality control processing was performed to remove peptides with insufficient data or LC-MS runs showing significant deviation from standard behavior . Peptides were normalized across the technical replicates using a rank invariant subset of peptides (p-value threshold of 0.1) followed by mean centering of the data based on the SPANS procedure .  Proteins were quantified using a standard R-Rollup method using the most abundant reference peptide, after filtering the peptides that were redundant, had low data content, or were outside the dominant significance pattern.  Comparative statistical analyses of time-matched mock with icSARS-Cov and icSARS-CoV ∆ORF 6 samples was performed using a Dunnett adjusted t-test to assess differences in protein average abundance, and a G-test to assess associations among factors due to the presence/absence of response.

1. Metz TO, Jacobs JM, Gritsenko MA, Fontes G, Qian WJ, et al. (2006) Characterization of the human pancreatic islet proteome by two-dimensional LC/MS/MS. J Proteome Res 5: 3345-3354.

2. Petyuk VA, Qian WJ, Hinault C, Gritsenko MA, Singhal M, et al. (2008) Characterization of the mouse pancreatic islet proteome and comparative analysis with other mouse tissues. J Proteome Res 7: 3114-3126.

3. Livesay EA, Tang K, Taylor BK, Buschbach MA, Hopkins DF, et al. (2008) Fully automated four-column capillary LC-MS system for maximizing throughput in proteomic analyses. Anal Chem 80: 294-302.

4. Eng JK, Mccormack AL, Yates JR (1994) An Approach to Correlate Tandem Mass-Spectral Data of Peptides with Amino-Acid-Sequences in a Protein Database. Journal of the American Society for Mass Spectrometry 5: 976-989.

5. Qian WJ, Liu T, Monroe ME, Strittmatter EF, Jacobs JM, et al. (2005) Probability-based evaluation of peptide and protein identifications from tandem mass spectrometry and SEQUEST analysis: the human proteome. J Proteome Res 4: 53-62.

6. Petritis K, Kangas LJ, Ferguson PL, Anderson GA, Pasa-Tolic L, et al. (2003) Use of artificial neural networks for the accurate prediction of peptide liquid chromatography elution times in proteome analyses. Anal Chem 75: 1039-1048.

7. Kiebel GR, Auberry KJ, Jaitly N, Clark DA, Monroe ME, et al. (2006) PRISM: a data management system for high-throughput proteomics. Proteomics 6: 1783-1790.

8. Jaitly N, Mayampurath A, Littlefield K, Adkins JN, Anderson GA, et al. (2009) Decon2LS: An open-source software package for automated processing and visualization of high resolution mass spectrometry data. BMC Bioinformatics 10: 87.

9. Monroe ME, Tolic N, Jaitly N, Shaw JL, Adkins JN, et al. (2007) VIPER: an advanced software package to support high-throughput LC-MS peptide identification. Bioinformatics 23: 2021-2023.

10. Zimmer JS, Monroe ME, Qian WJ, Smith RD (2006) Advances in proteomics data analysis and display using an accurate mass and time tag approach. Mass Spectrom Rev 25: 450-482.

11. Webb-Robertson BJ, McCue LA, Waters KM, Matzke MM, Jacobs JM, et al. (2010) Combined statistical analyses of peptide intensities and peptide occurrences improves identification of significant peptides from MS-based proteomics data. J Proteome Res 9: 5748-5756.

12. Matzke MM, Waters KM, Metz TO, Jacobs JM, Sims AC, et al. (2011) Improved quality control processing of peptide-centric LC-MS proteomics data. Bioinformatics 27: 2866-2872.

13. Webb-Robertson BJ, Matzke MM, Jacobs JM, Pounds JG, Waters KM (2011) A statistical selection strategy for normalization procedures in LC-MS proteomics experiments through dataset-dependent ranking of normalization scaling factors. Proteomics 11: 4736-4741.

14. Polpitiya AD, Qian WJ, Jaitly N, Petyuk VA, Adkins JN, et al. (2008) DAnTE: a statistical tool for quantitative analysis of -omics data. Bioinformatics 24: 1556-1558.
